# Direct Ectoine Production from Chitin Waste by a Wild-Type *Vibrio* Strain X511: Genomic Insights and Substrate Degradation

**DOI:** 10.3390/md24100362

**Published:** 2026-10-18

**Authors:** Qiaozhen Wang, Shushi Huang, Mei Lin, Chao Xu, Siming Liao, Youjiao Wu, Qiuyu Wang, Aoni Zhang, Ling Yang, Xiaoyun Ou, Feng Li

**Affiliations:** 1 National Key Laboratory of Non-Food Biomass Energy Technology, Guangxi Key Laboratory of Marine Natural Products and Combinatorial Biosynthesis Chemistry, Guangxi Academy of Sciences, Nanning 530007, China; 2 School of Food and Bioengineering, Changsha University of Science and Technology, Changsha 410005, China; 3 Guangxi Key Laboratory of Chemistry and Engineering of Forest Products, School of Chemistry and Chemical Engineering, School of Marine Sciences and Biotechnology, Guangxi Minzu University, Nanning 530006, China; 4 School of Chemistry and Chemical Engineering, Qiannan Normal University for Nationalities, Du yun 558000, China

**Keywords:** marine *Vibrio*, chitin waste, Ectoine, bioconversion, genomic analysis

## Abstract

Chitin is the second most abundant natural renewable biomass, widely present in seafood waste. Nevertheless, its crystalline structure makes processing difficult. Ectoine, a high value compatible solute, is common in pharmaceuticals and cosmetics. However, direct ectoine production from chitinous waste using wild-type microbial strains is rarely reported. Here, we show that *Vibrio diabolicus* X511 possesses a complete ectoine biosynthetic gene cluster (*ectABC-ask*) as well as native chitinolytic genes. These genomic features are consistent with the strain’s ability to directly ferment colloidal chitin, crab shell flakes, and shrimp shell powder for ectoine production, eliminating the need for exogenous chitinase supplementation. With 5 g/L yeast extract as the nitrogen source, strain X511 produced 2.172 g/L ectoine from colloidal chitin, a titer comparable to, or even higher than, those reported for several wild-type strains grown on soluble carbon sources. Using highly crystalline crab shell flakes and raw shrimp shell powder as substrates, the strain achieved titers of 0.259 g/L and 0.181 g/L respectively, accompanied by substrate weight losses of 60% and 43.47%. FESEM, XRD, and FTIR analyses confirmed the strain’s capacity to effectively disrupt crystalline structure of chitin and degrade raw shrimp shell biomass. These findings suggest that X511 may be a promising biocatalyst for the effective bioconversion of chitinous waste to high value chemicals.

## 1. Introduction

Chitin, the second most abundant natural polymer after cellulose, consists of N-acetylglucosamine (GlcNAc) residues linked by β-1,4-glycosidic bonds [1,2]. In shrimp and crab processing, 60–80% of the raw material is discarded as waste, with the edible meat comprising only 20–40% [3]. With the expanding global shrimp and crab market, generating 6–8 million tons of shellfish waste each year from food processing [4]. Traditional waste disposal methods, including incineration, landfilling, and ocean dumping, cause severe environmental pollution [5]. Therefore, the efficient utilization and high value conversion of seafood waste have drawn considerable research attention. These waste shells contain approximately 20–40% protein, 20–50% calcium carbonate, and 15–40% chitin [4]. Among these components, chitin, a major constituent of shrimp shell waste, has attracted particular attention due to its unique polymeric structure and broad applicability in biomaterials, pharmaceuticals, and food products [6,7,8,9].

Chitin is structurally similar to cellulose, differing primarily in the substitution of a hydroxyl group at the C-2 position with an acetylamino (–NHCOCH_3_). This acetamide functionality enables the synthesis of nitrogen-containing platform molecules, which are inaccessible to lignocellulosic biomass [10]. Although chitin is an abundant nitrogen rich biopolymer, its low solubility in water and most organic solvents restricts its processing and application [11]. Chemical degradation, on the other hand, introduces separate issues, including environmental concerns and unsatisfactory product purity [12,13,14]. In recent years, bioconversion has become a viable route for the valorization of shrimp shells and other shellfish chitin waste into valuable enzymes and bioactive materials. On the one hand, the utilization of chitin waste focuses on the separation and recovery of native components from shrimp shells. For example, solid-state fermentation of shrimp shell waste using *Streptomyces* sp. SCUT-3, followed by lactic acid treatment, yielded 33.6 g of free amino acids, 16.7 g of oligopeptides, 7.4 g of calcium (in the form of calcium lactate), and 19.5 g of chitin from just 100 g of raw shells [15]. On the other hand, another approach employs shrimp shell powder as a substrate for microbial production of chitinolytic and proteolytic enzymes. Alternatively, it can be used to obtain bioactive oligosaccharides through microbial degradation. For instance, using chitin powder and shrimp shell powder as substrates, a two-stage enrichment process yielded a microbial consortium, designated XHQ10, with chitinase and LPMO activities of 1.46 U/mL and 54.62 U/mL, respectively, which produced COS with a DP > 10 that exhibited excellent antioxidant activity [16]. Although significant progress has been made in the bioconversion of shrimp shell waste, the direct conversion of untreated chitinous waste into ectoine remains largely unexplored.

Ectoine, a cyclic amino acid derivative also known as 1,4,5,6-tetrahydro-2-methyl-4 -pyrimidinecarboxylic acid, is widely found in halophilic microorganisms, where it functions as a compatible solute and an osmoprotectant. This compound maintains the native conformation of biological macromolecules and safeguards cells, membranes, proteins, enzymes, and nucleic acids from damage under extreme environmental conditions, such as high osmolarity, elevated temperature, and freeze–thaw cycles [17,18]. Ectoine, owing to its unique protective mechanism, holds broad application prospects in biotechnology, cosmetics, and pharmaceuticals. It has been explored for skin protection, anti-aging, and cell damage prevention [19], as well as for addressing Alzheimer’s disease [20] and mitigating inflammatory responses [21,22]. Ectoine commands a global annual demand of approximately 15,000 tons and a retail price of roughly 1000 USD/kg, which places it among the typical high value added products [23]. Glucose, sodium glutamate, and sucrose are the most common carbon sources for ectoine production [24,25,26,27]. Among these, metabolically engineered strains cultivated on glucose have delivered titers as high as 131.8 g/L [26]. However, the high cost of pure carbon sources remains a major bottleneck for industrial scale production, this highlights the urgent need for low cost alternatives. Recent research has increasingly focused on using domestic, agricultural, and industrial wastes as low cost carbon and nitrogen sources for ectoine production, an approach that serves both environmental remediation and resource valorization. Specifically, corn protein hydrolysate, an agricultural byproduct rich in aspartate and glutamate, has served as a dual carbon and nitrogen source for ectoine production [28]. Methane from various industrial sources has also been explored as a carbon substrate for this purpose [29]. In another development, an electro-biocatalytic system was designed to convert CO_2_ into CH_4_ through electrocatalytic reduction. The generated CH_4_ then served as an electrofuel to support the engineered methanotrophic bacterium *Methylomicrobium alcaliphilum* 20Z for ectoine synthesis [30], the search for cheaper and more sustainable feedstocks continues. Shrimp shell waste, as an inexpensive and renewable natural biomass resource, remains relatively underexplored as a fermentation feedstock for ectoine production.

Members of the family *Vibrionaceae* are well recognized as important chitin degraders in marine environments [31,32]. Strain X511, a marine *Vibrio* isolate, can degrade multiple polysaccharides, including alginate [33,34] and chitin. These features make strain X511 a suitable candidate for ectoine production from chitinous wastes. To evaluate this potential, colloidal chitin was used as a low-crystallinity, loosely structured, and relatively pure chitin substrate for model studies. Crab shell flakes, which had been deproteinized and demineralized, were used as a representative of highly crystalline α-chitin. Shrimp shell powder was used as a representative chitinous waste to assess practical applicability.

In this study, we analyzed the genomic information of the wild-type marine strain X511 to evaluate its enzymatic potential for chitin utilization. Liquid fermentation was conducted using colloidal chitin, commercially obtained crab shells, and untreated shrimp shell waste as substrates. Through preliminary optimization of fermentation conditions, we evaluated the strain’s ability to produce ectoine from colloidal chitin. Solid-state analytical techniques including SEM, XRD, and FTIR were further used to elucidate the distinctive degradation mechanisms of strain X511 toward highly crystalline α-chitin and raw shrimp shells. Importantly, this study directly converts chitinous feedstock into ectoine using a wild-type strain, by passing preliminary enzymatic hydrolysis and addition of exogenous chitinases. This distinguishes the present work from previous studies that relied on purified carbon sources or enzymatically pretreated substrates. This work provides both a theoretical basis and practical insights for the efficient bioconversion of shrimp shell waste into valuable products.

## 2. Results

### 2.1. Genomic Analysis

#### 2.1.1. Gene Functional Annotation of Strain X511

**Figure 1:**
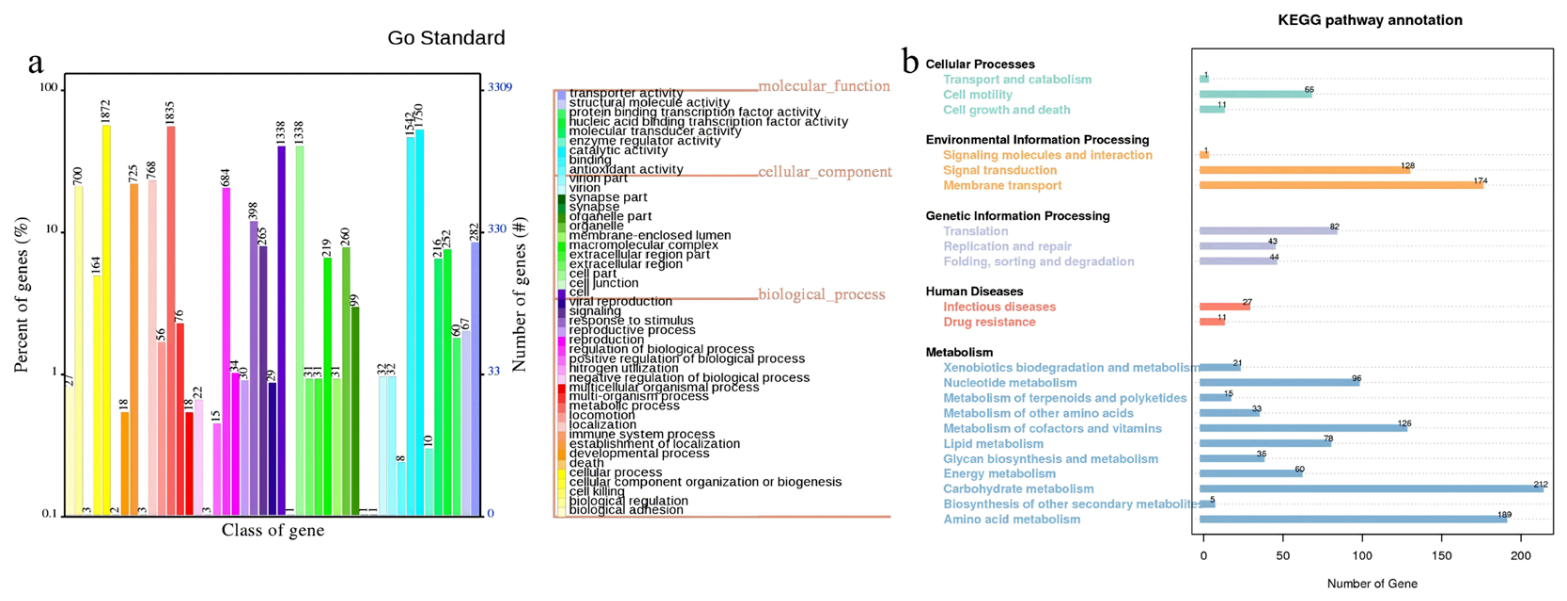
Genome annotation results for strain X511. (**a**) Bar chart of categorized statistics of GO database annotation results; (**b**) KEGG database annotation result categorization statistics histogram. The symbol “#” in the y-axis label is used as a shorthand for “number” and indicates the number of genes.

Genome sequencing of strain X511 yielded a total size of 5.16 Mbp, with 30 contigs and a G + C content of 44.81 mol%. Functional annotation was performed using the NR, Swiss-Prot, COG, GO, and KEGG databases. A total of 3308 genes (69.89% of the total) were assigned GO terms, while 2833 genes (59.86%) were mapped to KEGG pathways (Table 1). The annotation results for GO and KEGG are summarized in Figure 1a and Figure 1b, respectively. Among the GO categories, genes involved in biological processes were the most abundant (7718, 50.4%), followed by those related to molecular function (4187, 27.3%) and cellular component (3414, 22.3%). KEGG analysis further showed that carbohydrate metabolism and amino acid metabolism represented the most enriched pathways in the genome. CAZy annotation revealed 116 carbohydrate active enzymes in the strain X511 genome, including 46 glycoside hydrolases, 26 glycosyltransferases, 25 carbohydrate-binding modules, nine polysaccharide lyases, five carbohydrate esterases, and five auxiliary oxidases (Table 2). Among these, several glycoside hydrolases were identified as potential chitin degrading enzymes, as described below. The genome sequence of strain X511 has been deposited in GenBank under accession number JCCFFL000000000, where it is classified as *Vibrio diabolicus*.

#### 2.1.2. Genetic Basis for Chitin Utilization

**Figure 2:**
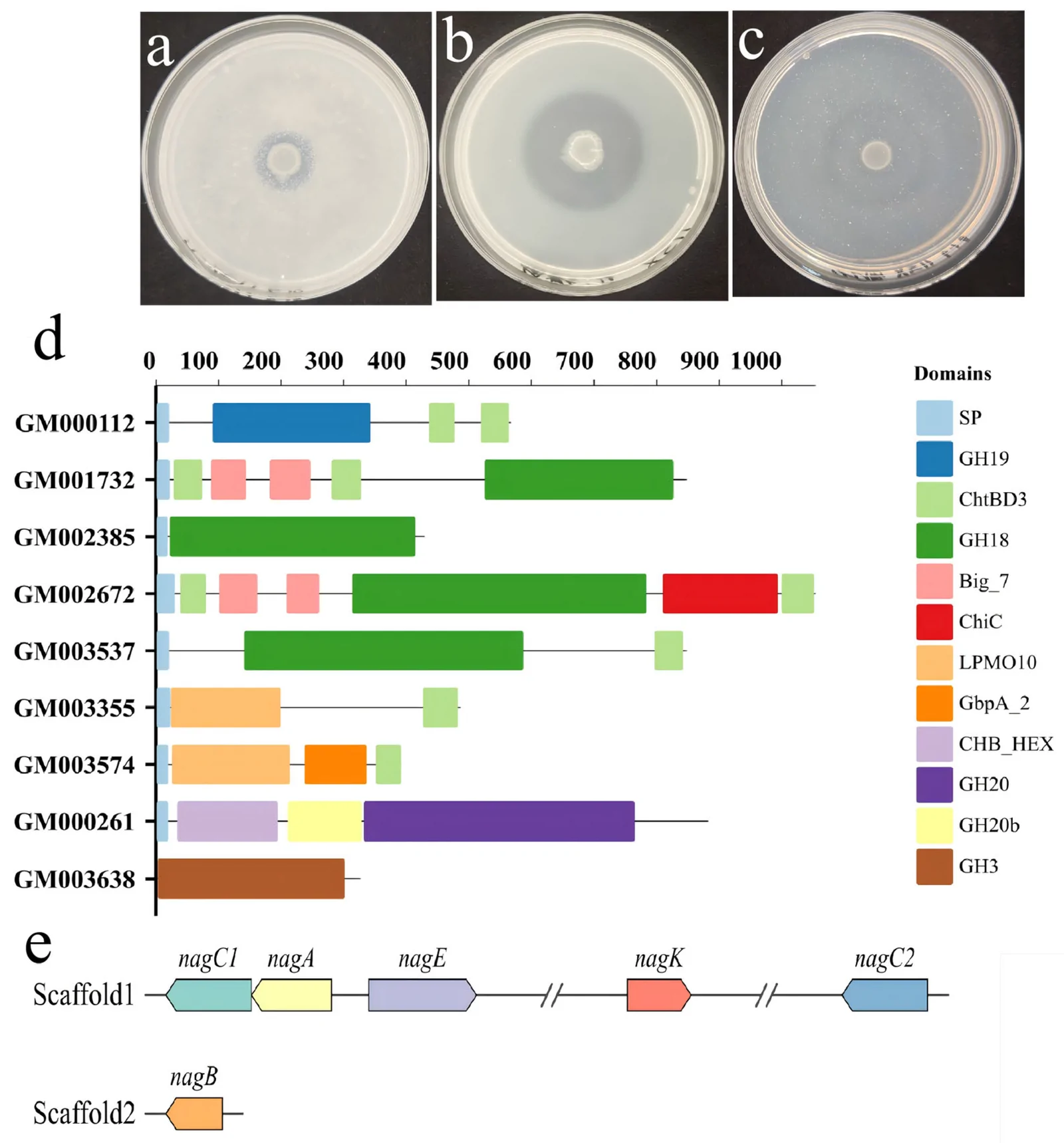
Molecular machinery of shrimp shell utilization by strain X511. (**a**–**c**) Extracellular hydrolase activities on agar plates supplemented with colloidal chitin (**a**), skim milk (**b**), and Tween 80 (**c**), clear zones indicate hydrolysis of chitin and casein, whereas opaque precipitation indicates esterolysis; (**d**) conserved domain architectures of chitinolytic enzymes identified in the genome; SP, signal peptide; GH18/GH19/GH20/GH20b/GH3, glycoside hydrolase families 18, 19, 20, 20b, and 3; ChtBD3, chitin-binding domain 3; Big _7, bacterial immunoglobulin-like domain; ChiC, chitinase C; LPMO10, lytic polysaccharide monooxygenase family 10; GbpA_2, N-acetylglucosamine binding protein domain 2; CHB-HEX, chitobiase/β-hexosaminidase N-terminal domain. (**e**) Genetic organization of the GlcNAc utilization gene cluster. *nagE, nagA, nagB, nagK,* and *nagC* encode PTS permease, GlcNAc-6-P deacetylase, GlcN-6-P deaminase, GlcNAc kinase, and ROK family regulator, respectively. Arrows indicate the direction of transcription. Detailed domain and gene annotations are provided in the text.

Plate-based enzyme activity assays demonstrated that strain X511 could degrade chitin, protein, and lipid substrates (Figure 2a–c), suggesting an enzymatic basis for direct conversion of shrimp shell waste. Genomic annotation further revealed the molecular architecture of its chitinolytic system, with nine genes identified as encoding chitin degrading enzymes. They are four GH18 family chitinases, one GH19 family chitinase, one GH20 family and one GH3 family β-N-acetylglucosaminidase (NAGase), and two AA10 family lytic polysaccharide monooxygenases (LPMO10s) (Figure 2d). Except for the GH3 family NAGase (GM003638), all of these enzymes contain one or two chitin binding domains (ChtBD3) in their amino acid sequences, and all were predicted to contain signal peptides.

Genes encoding GlcNAc utilization enzymes were also identified in the strain X511 genome, including *nagE* (PTS permease), *nagA* (N-acetylglucosamine-6-phosphate deacetylase), *nagB* (glucosamine-6-phosphate deaminase), *nagK* (N-acetylglucosamine kinase), and *nagC* (ROK family transcriptional regulator). Among them, *nagC1*, *nagA*, and *nagE* are organized as a cluster, whereas *nagK*, *nagC2*, and *nagB* are located in separate genomic regions (Figure 2e). This genomic organization suggests that the strain may possess a complete pathway for GlcNAc uptake and metabolism.

#### 2.1.3. Synthesis and Degradation Pathways of Ectoine in Strains X511

**Figure 3:**
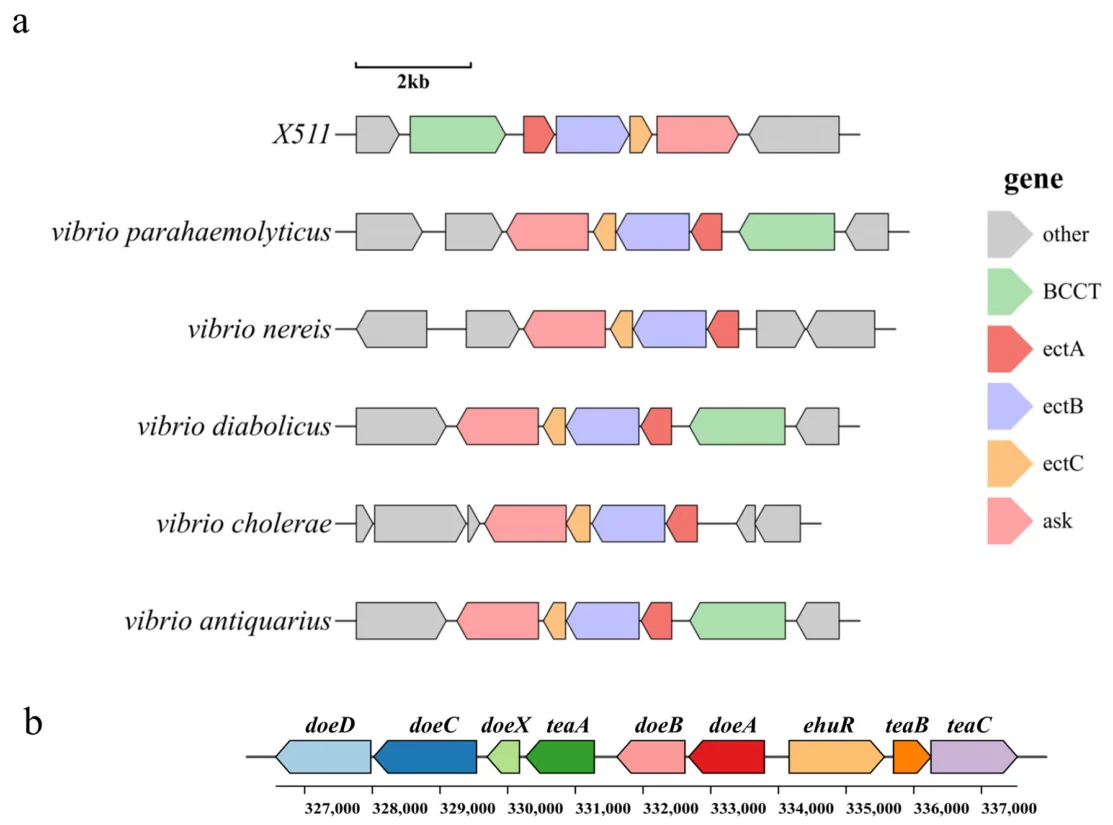
Genetic organization of the ectoine biosynthesis and degradation gene clusters in strain X511. (**a**) Comparison of the ectoine biosynthesis gene clusters, comprising the core biosynthetic genes (*ectABC-ask*) and a BCCT family transporter; (**b**) organization of the ectoine degradation gene cluster. *ectA*, *ectB*, *ectC*, and *ask* encode L-2,4-diaminobutyrate acetyltransferase, diaminobutyrate aminotransferase, ectoine synthase, and aspartate kinase, respectively; *doeA*, *doeB*, *doeC*, and *doeD* encode ectoine hydrolase, N-α-acetyl-L-2,4-diaminobutyrate deacetylase, aspartate-semialdehyde dehydrogenase, anddiaminobutyrate aminotransferase, respectively; *doeX*, encoding ectoine degradation regulator; *ehuR*, encoding ectoine utilization regulator; *teaA*–*teaC*, encoding TRAP-type ectoine transporter genes. Arrows indicate the direction of transcription.

Strain X511 is a halotolerant Vibrio strain capable of surviving in high salt environments [34], suggesting the presence of osmotic adaptation systems in its genome. A full ectoine biosynthetic gene cluster was identified (Figure 3a), with the conserved *ectABC-ask* structure also found in *V. parahaemolyticus*, *V. nereis*, and *V. diabolicus*. This cluster includes *ectA*, *ectB*, and *ectC* for ectoine synthesis, as well as *ask*, which encodes aspartate kinase, contributing to the supply of the ectoine precursor L-aspartate-β-semialdehyde. A complete catabolic gene cluster was also detected in a separate region (Figure 3b), containing *doeABCDX*, *teaA*, and *eutRBC*. These genes encode an ectoine hydrolase (DoeA), a TRAP-type transporter (TeaA), and regulatory factors (EutR and EutC), which together govern ectoine catabolism and transport.

### 2.2. Ability of Strain X511 for Ectoine Production from Colloidal Chitin

#### 2.2.1. Effect of NaCl Concentration on Ectoine Production from Colloidal Chitin

**Figure 4:**
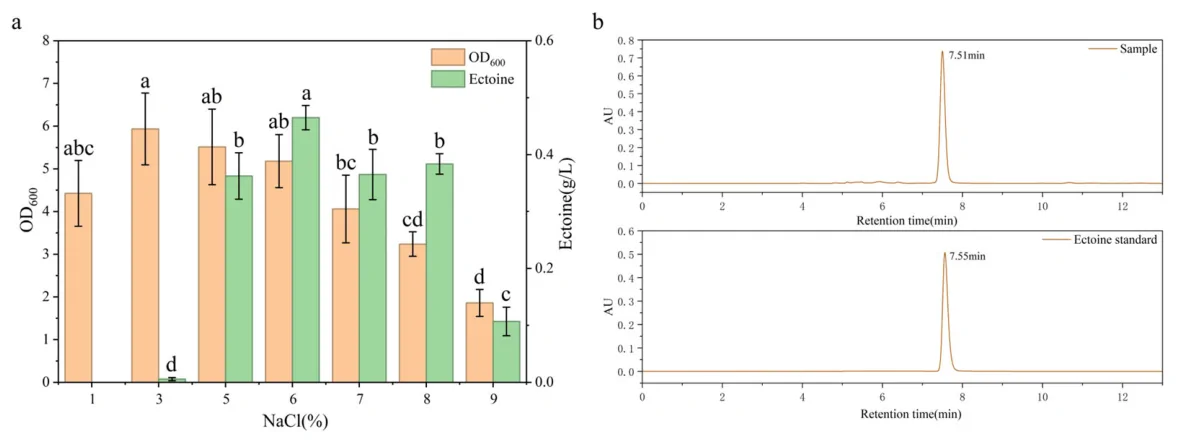
Effect of NaCl concentration on ectoine production from colloidal chitin by strain X511. (**a**) Cell growth and ectoine production of strain X511 cultivated with colloidal chitin as the sole carbon source at various NaCl concentrations. (**b**) HPLC chromatograms of the ectoine standard and the ectoine extract from strain X511. Different lowercase letters indicate significant differences (*p* < 0.05).

When colloidal chitin served as the sole carbon source, ectoine production by strain X511 displayed a clear dependence on salt concentration. At low salinity (1–3% NaCl), ectoine was barely detectable. The titer rose sharply at 5% NaCl and peaked at 0.489 g/L with 6% NaCl, then dropped steeply to 0.107 g/L at 9% NaCl (Figure 4a). HPLC analysis confirmed the fermentation product as ectoine, with the major peak showing a retention time identical to that of the standard (Figure 4b). This result agrees with the genomic data, where no *ectD* homolog was found, suggesting that the biosynthetic pathway in X511 terminates at ectoine rather than its hydroxylated derivatives.

#### 2.2.2. Potential for Enhanced Ectoine Production from Colloidal Chitin by Strain X511

**Figure 5:**
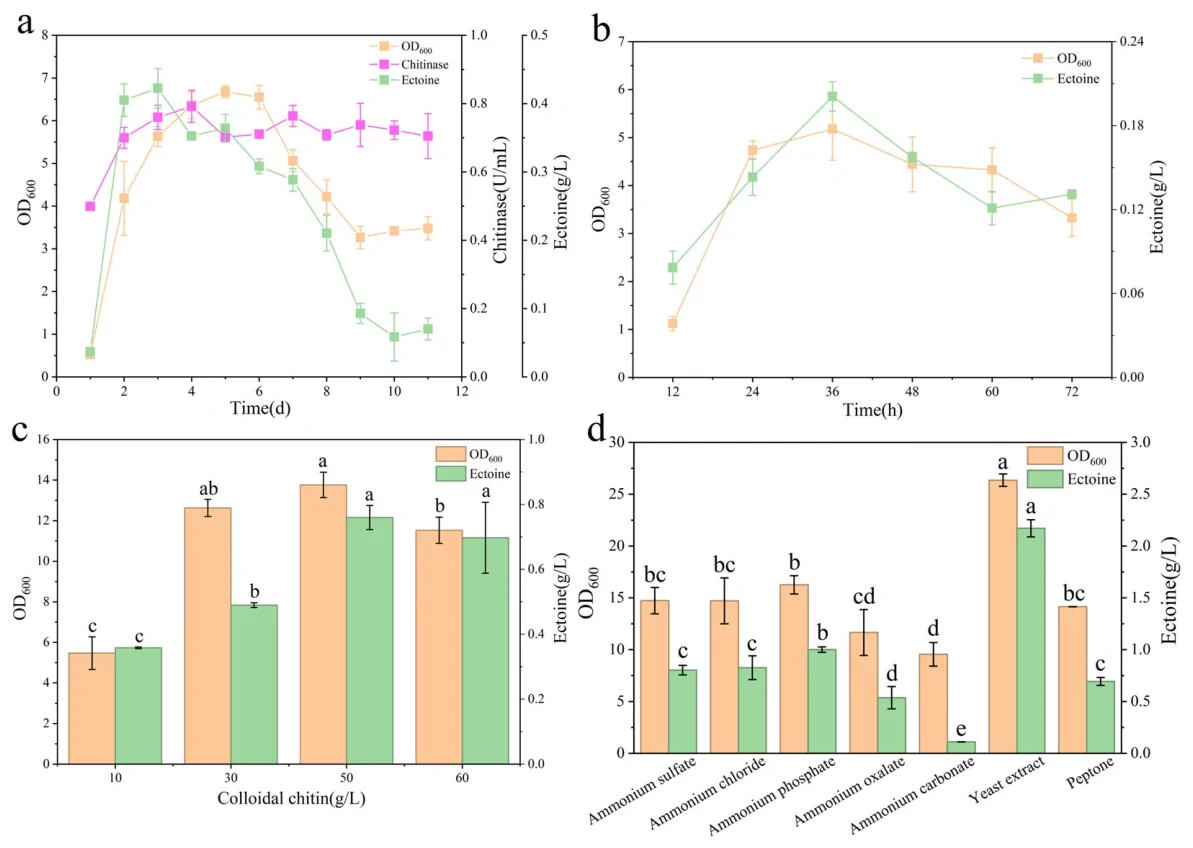
Optimization of ectoine production by strain X511. (**a**) Time course of biomass (OD_600_), chitinase activity, and ectoine production on colloidal chitin; (**b**) time course of biomass (OD_600_) and ectoine production on GlcNAc; (**c**) ectoine production at different colloidal chitin concentrations; (**d**) ectoine production with different nitrogen sources. Different lowercase letters indicate significant differences (*p* < 0.05).

During fermentation of colloidal chitin by strain X511, key parameters were monitored to understand the interplay between substrate degradation and ectoine formation. The ectoine titer reached 0.423 g/L after 3 days of fermentation, then dropped to 0.364 g/L after 5 days and continued to decline with extended fermentation (Figure 5a). After ectoine production peaked, Chitinase activity remained relatively stable throughout the process, ranging from 0.70 to 0.79 U/mL. Although ectoine production decreased after 3 days, cell biomass (OD_600_) continued to increase and peaked after 5 days (Figure 5a). When GlcNAc was supplied as the sole carbon source, the highest ectoine yield of 0.201 g/L was obtained after 36 h of fermentation (Figure 5b), indicating that strain X511 could directly utilize the chitin monomer for ectoine production. Based on this, part of the carbon flux from chitin metabolism is proposed to be channeled toward ectoine synthesis.

The carbon source concentration and nitrogen source were optimized to further explore the potential of strain X511 for ectoine production from colloidal chitin (Figure 5c,d). When the colloidal chitin concentration ranged from 10 to 50 g/L, ectoine production increased progressively with rising carbon source levels, showing a positive correlation. The highest ectoine titer of 0.760 g/L was obtained at 50 g/L, where biomass also reached its maximum; therefore, 50 g/L was selected as the optimal concentration. However, further increasing the carbon source to 60 g/L led to a decline in both biomass and ectoine production (0.697 g/L), suggesting that cell growth began to be inhibited under higher carbon source concentrations (Figure 5c).

Under optimized carbon source conditions, the effects of different nitrogen sources on ectoine production were evaluated (Figure 5d). Among the seven sources tested, including ammonium sulfate, ammonium chloride, ammonium phosphate, ammonium oxalate, ammonium carbonate, yeast extract and peptone, yeast extract produced the highest ectoine titer at 2.172 g/L and also supported the greatest biomass accumulation, significantly outperforming all other treatments (*p* < 0.05). This indicates that yeast extract supplementation was particularly favorable for ectoine biosynthesis. Among the inorganic nitrogen sources, ammonium phosphate proved to be the most effective, yielding 1.001 g/L ectoine, followed by ammonium chloride at 0.826 g/L and ammonium sulfate at 0.802 g/L. Compared with the pre-optimization titer of 0.423 g/L, the ectoine titer was increased approximately 5.1-fold through the optimization of carbon and nitrogen sources alone, highlighting the considerable potential of strain X511 for ectoine production from colloidal chitin.

### 2.3. Ectoine Production from Crab Shell Flakes by Strain X511

**Figure 6:**
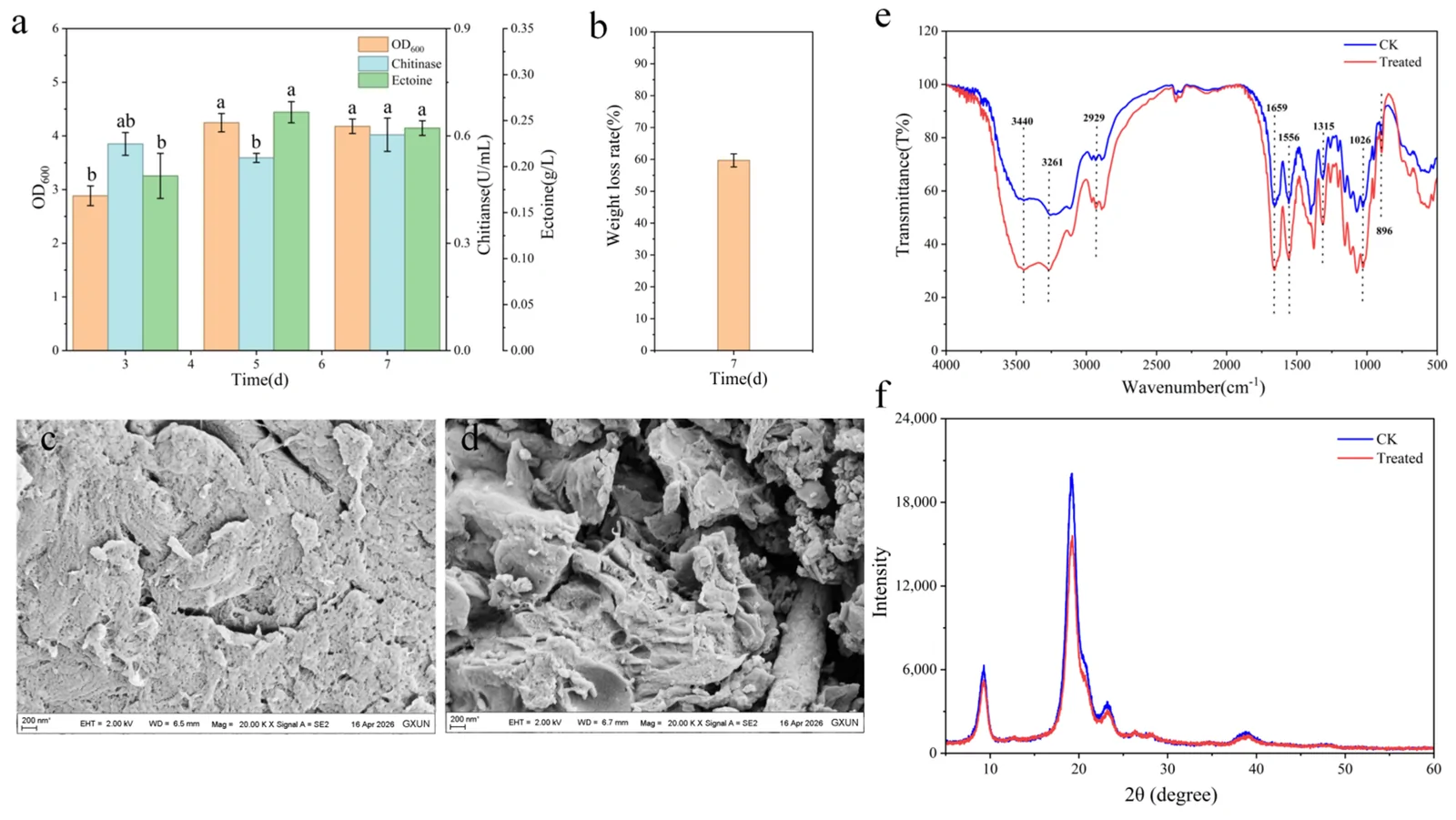
Degradation of crab shells by strain X511. (**a**) Time course of biomass, chitinase activity, and ectoine production; (**b**) weight loss rate after 7 days; (**c**,**d**) FESEM images of untreated (**c**) and treated (**d**) crab shells at ×20,000 magnification; (**e**) FTIR spectra and (**f**) XRD patterns of untreated (blue) and treated (red) crab shell. Different lowercase letters indicate significant differences (*p* < 0.05). The symbol “*” next to the scale bar was automatically generated by the FESEM instrument and has no special meaning.

Using crab shell flakes as the sole carbon source, strain X511 produced 0.259 g/L ectoine after 5 days fermentation, which slightly decreased to 0.242 g/L after 7 days. Biomass peaked at the same time point and then entered a stationary phase. Chitinase activity remained within a narrow range at 0.539–0.603 U/mL throughout fermentation (Figure 6a), although some time points differed significantly (*p* < 0.05). The shell flakes lost 60% of their weight after 7 days (Figure 6b).

Strain X511 exhibited considerable degradative activity against highly crystalline α-chitin. To better understand its mode of action, the structural impact of X511 treatment on crab shell flakes was examined using FESEM. The untreated α-chitin showed a dense, ordered structure with a relatively intact surface (Figure 6c). Following 7 days of treatment, however, this highly crystalline and compact architecture was clearly disrupted, as evidenced by surface cracking, fragmentation, visible erosion pits, and roughened areas (Figure 6d). FTIR spectra of the untreated and X511 treated crab shell flakes displayed identical characteristic peaks (Figure 6e). The typical absorption bands of α-chitin were observed at 897 cm^−1^, 1315 cm^−1^, 1556 cm^−1^ and 1659 cm^−1^ [35,36], indicating that the strain treatment did not alter the major chemical composition of the crab shell flakes. After 7 days of treatment with X511, the intensities of the infrared absorption bands at 3440 cm^−1^, 3261 cm^−1^, 2929 cm^−1^, 1659 cm^−1^, 1556 cm^−1^ and 1026 cm^−1^ were significantly enhanced. XRD analysis was further performed to evaluate possible changes in crystallinity. The characteristic α-chitin diffraction peaks at approximately 9.13° and 19.19° were clearly observed in the XRD pattern of crab shell chitin (Figure 6f). After 7 days of treatment with strain X511, a marked decrease in peak intensities was detected, pointing to crystalline disruption and reduced crystallinity.

### 2.4. Ectoine Production from Shrimp Shell Waste by Strain X511

**Figure 7:**
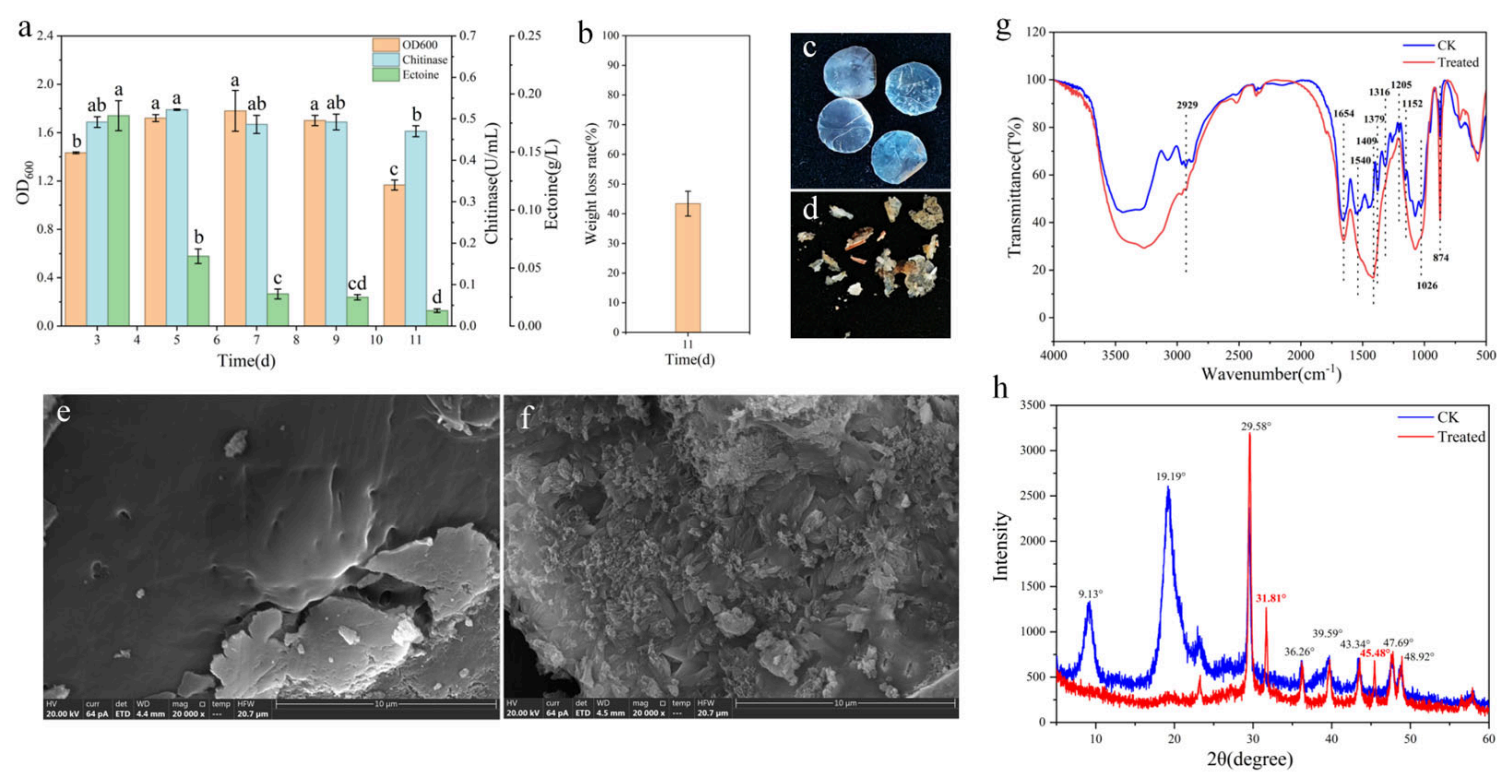
Degradation of shrimp shell powder by strain X511. (**a**) Time course of biomass, chitinase activity, and ectoine production; (**b**) weight loss rate after 11 days. (**c**,**d**) Photographs of shrimp shell flakes without (**c**) and with (**d**) the strain for 5 days. (**e**,**f**) FESEM images of untreated (**e**) and treated (**f**) samples after 11 days (×20,000). (**g**) FTIR spectra and (**h**) XRD patterns of untreated (blue) and treated (red) samples after 11 days. Different lowercase letters indicate significant differences (*p* < 0.05).

Using untreated shrimp shell powder as the sole carbon source, strain X511 achieved a peak ectoine titer of 0.181 g/L after 3 days fermentation, which then dropped sharply to 0.06 g/L after 5 days and continued to decline with prolonged fermentation (Figure 7a). Throughout the entire fermentation period, chitinase activity remained relatively stable at 0.467–0.522 U/mL. After fermentation, the weight loss of shrimp shell powder reached 43.47% (Figure 7b), and most of the shell flakes had disintegrated into fragments after 5 days treatment (Figure 7c,d).

FESEM analysis of shrimp shell powder surface morphology provided further evidence of degradation by strain X511. While the untreated shells showed a smooth and intact surface (Figure 7e), those treated with strain X511 exhibited a roughened surface with visible pores, as well as flower-like crystalline structures (Figure 7f), indicating structural disruption of the shell matrix. FTIR spectra further revealed notable differences between the treated and untreated samples (Figure 7g). Several characteristic peaks observed in the control group, including those at 1152, 1205, 1316, 1379, and 1540 cm^−1^, were absent in the treated sample, suggesting substantial structural changes in the shell powder. Meanwhile, the peaks at 1026, 1654, and 2929 cm^−1^ exhibited reduced intensities, whereas the peaks at 874 and 1409 cm^−1^ showed marked enhancement. XRD analysis revealed that after 11 days of treatment with strain X511, the characteristic chitin diffraction peaks of shrimp shell powder at 9.13° and 19.19° (2θ) were no longer detectable. In parallel, the peaks at 19.58° was enhanced, the peaks at 39.59°, 43.34°, and 47.69° (2θ) became narrower, while new peaks emerged at 31.81° and 45.48° (2θ) (Figure 7h).

### 2.5. NAGase Activity

During ectoine fermentation on chitinous substrates, strain X511 exhibited consistently low NAGase activity. In the colloidal chitin group, it was 15.11 mU/mL at 5 days and 18.46 mU/mL at 7 days, with a significant difference (*p* < 0.05). The NAGase activities in the crab shell flake and shrimp shell powder groups remained within narrow ranges of 10.07–11.08 mU/mL and 4.23–4.78 mU/mL, respectively (Table 3). NAGase activities in all substrate groups were significantly lower than the corresponding chitinase activities (Figure 5a, Figure 6a and Figure 7a), while the chitinase activities remained within narrow ranges throughout the fermentation period.

### 2.6. Proposed Metabolic Pathway for Shrimp Shell Conversion to Ectoine

**Figure 8:**
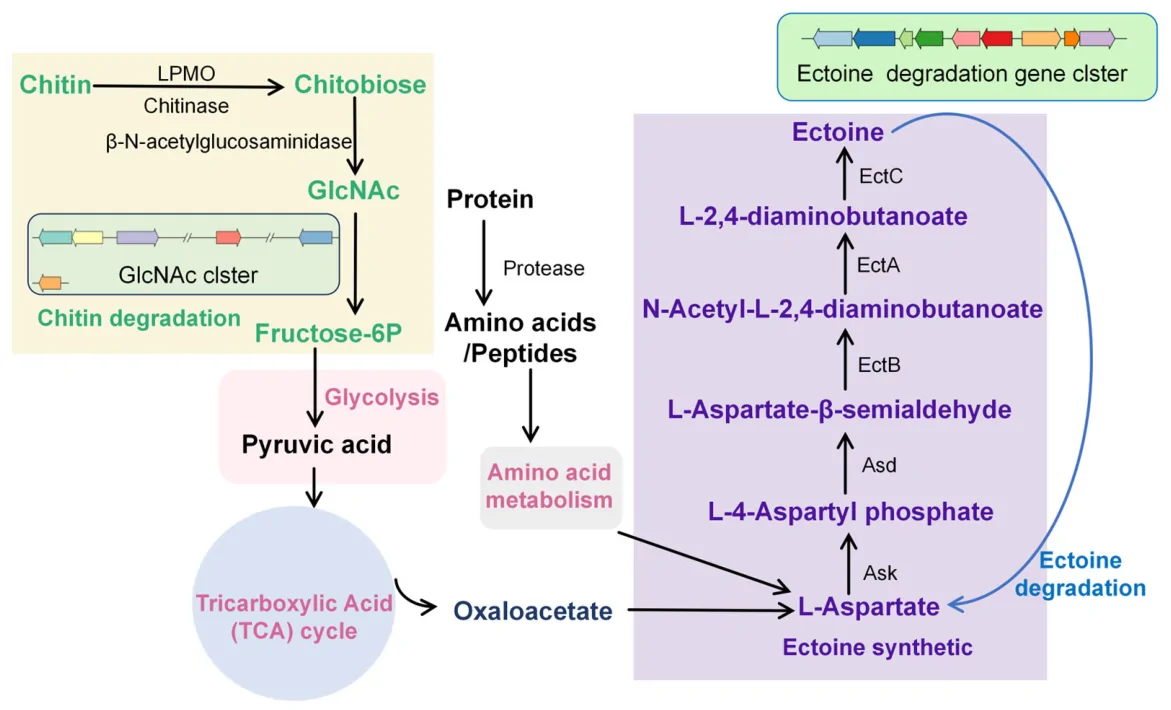
Proposed metabolic pathway for ectoine biosynthesis from shrimp shell powder by strain X511. GlcNAc, N-acetylglucosamine; Ask, Aspartokinase; Asd, aspartate-semialdehyde dehydrogenase; EctA, diaminobutyrate acetyltransferase; EctB, diaminobutyrate-2-oxoglutarate transaminase; EctC, ectoine synthase.

Based on genomic analysis and phenotypic experimental data, a potential metabolic pathway was deduced for strain X511 to utilize shrimp shell powder for ectoine synthesis (Figure 8). Shrimp shell waste is a natural composite composed primarily of protein, chitin, and calcium carbonate, which are closely associated with one another. Strain X511 is predicted to possess an extracellular enzyme system capable of directly acting on this waste material. It is proposed that secreted proteases hydrolyze the protein components first, thereby exposing the embedded chitin. The secreted LPMOs and chitinases are then proposed to act synergistically on the crystalline chitin, increasing the exposure of amorphous regions and facilitating the binding of chitinases to the loosened substrate. This concerted action may lead to cleavage of β-1,4-glycosidic bonds and generates chitooligosaccharides along with GlcNAc monomers. The resulting chitooligosaccharides are presumably further degraded into GlcNAc by NAGases from the GH20 and GH3 families. Once released, GlcNAc is proposed to be taken up by the cells and channeled through intracellular enzymatic cascades, ultimately leading to ectoine biosynthesis. This proposed pathway provides a framework for understanding how strain X511 may convert shrimp shell waste into ectoine, but further experimental validation is required.

### 2.7. Comparison of Ectoine Production with Other Wild-Type Strains

In recent years, the production of ectoine from low-cost biomass hydrolysates has drawn growing interest, but wild-type strains generally produce ectoine at low yields. As summarized in Table 4, most of the reported wild-type strains with high ectoine titers rely on soluble carbon sources such as glucose, glutamate, or biomass hydrolysates. These substrates are readily accessible to the cells. In contrast, strain X511 was cultivated on insoluble polymeric substrates including colloidal chitin, crab shell flakes, and shrimp shell powder, without pretreatment or the addition of exogenous chitinases. Notably, despite utilizing insoluble polymeric chitinous substrates, the ectoine titer achieved by strain X511 on colloidal chitin was comparable to or higher than that of many wild-type strains grown on soluble substrates (Table 4). Therefore, the advantage of X511 lies not only in the ectoine titer achieved but also in its ability to directly utilize polymeric chitinous feedstock.

## 3. Discussion

The genome of strain X511 contains numerous genes that encode enzymes involved in the degradation of complex polysaccharides. Previous studies have confirmed its strong ability to utilize complex carbohydrates. It efficiently produces alginate lyase, an enzyme that converts alginate or kelp into bioactive alginate oligosaccharides. These oligosaccharides can subsequently be converted into butanediol, an important industrial compound [33,34], highlighting its considerable potential for adding value to marine biomass. Genomic analysis revealed the presence of two LPMO10s, together with multiple GH18/GH19 chitinases and GH20/GH3 NAGases, indicating that strain X511 possesses a comprehensive enzymatic system for chitin degradation. The chitin binding domains (ChtBD3) found in these enzymes are proposed to anchor the enzymes to the chitin surface, thereby facilitating the catalytic domains in their efficient breakdown of insoluble chitin [41]. According to their cleavage mode, most AA10 members belong to the C1-oxidizing category and function as copper-dependent oxidases targeting the C1-H bonds of crystalline chitin. The oxidative modification reshapes the substrate surface and exposes additional chain termini, which facilitates the binding and activity of chitinases [42,43]. Based on this, LPMO-mediated oxidative cleavage is proposed as a possible mechanism in strain X511. Taken together, genomic and physicochemical evidence suggests that strain X511 possesses a chitinolytic system capable of degrading crystalline chitin. Chitin breakdown is proposed to generate GlcNAc oligomers that are subsequently metabolized intracellularly. In contrast to the tight and compact organization of the *nag* regulon in *Escherichia coli*, the genetic arrangement for GlcNAc catabolism in *Vibrio* species tends to be more dispersed and complex, as reflected in the fact that *nagE* and *nagAC* form an operon while *nagB* is located elsewhere [44,45]. Collectively, these core genes are predicted to form a complete GlcNAc utilization pathway, providing a metabolic basis for chitin degradation and the subsequent channeling of its carbon skeleton toward the biosynthesis of high-value compounds. It should be noted that the individual roles of these enzymes, LPMO-mediated oxidative cleavage, and the intracellular conversion of chitin-derived carbon are proposed based on genomic evidence. Direct experimental confirmation, such as enzyme functional assays and detection of oxidized products, was not obtained in this study.

In many microorganisms, the ectoine and hydroxyectoine biosynthetic gene cluster *ectABCD* is frequently followed by an *ask* gene (*ask_ect*). Strain X511 possesses a typical *ectABC-ask* structure, with Ask_Ect serving as a specialized enzyme for this osmoadaptative pathway. Studies in *E. coli* have shown that strains coexpressing the *ectABCD-ask* cluster produced significantly higher ectoine/hydroxyectoine titers than strains expressing the *ectABCD* cluster alone [46]. Aspartate kinase was identified as a key target for overcoming the bottleneck in ectoine biosynthesis in metabolic engineering studies. Coexpression of the rate-limiting enzyme mutant EctB (E407D) and an aspartate kinase mutant in *E. coli* resulted in a 140% increase in ectoine production [47]. These studies indicate that the *ectABC-ask* architecture represents a widely conserved osmoadaptation strategy in halophilic and halotolerant bacteria, enabling these strains to cope with fluctuating salinity environments.

Ectoine synthesis in strain X511 was strongly dependent on salt stress, and a moderate salinity level was required for efficient production. This dependence is consistent with previous observations that the *ectABC* genes are upregulated in response to salt induction in other strains [48]. The substantial increase in ectoine production at 6% NaCl suggests that the *ectABC-ask* gene cluster may be efficiently activated under this salinity condition. However, when salinity was raised to 7–9%, both biomass and ectoine production decreased, likely due to the adverse effects of extremely high salt concentrations on cellular functions [49]. CosR has been identified as a global repressor of multiple compatible solute systems in *Vibrio* species. It directly suppresses the expression of operons such as *ectABC* and *betIBA* under low salt conditions, with *ectA* downregulated by 794.6 fold and *asp_ect* by 204.9 fold [50]. At low NaCl concentrations (1–3%), strain X511 produced barely detectable levels of ectoine, suggesting that a CosR-like regulatory mechanism may govern its osmoadaptation strategy. For microorganisms, the utilization of complex or non-optimal carbon sources imposes additional metabolic burdens, which may compromise their ability to cope with other environmental stresses such as osmotic pressure [51,52]. Notably, strain X511 maintained its osmoregulatory capacity when grown on the complex insoluble polymer colloidal chitin as the sole carbon source, thus laying a foundation for coupling chitin valorization with high salt fermentation.

The effect of carbon source on ectoine production has been investigated in other species. Under optimized conditions, *Nesterenkonia xinjiangensis* achieved a maximum ectoine titer of 1.231 g/L after 36 h of fermentation with sucrose as the carbon source [25]. The engineered strain *Halomonas campaniensis* XH26 yielded an average peak ectoine titer of 1.798 g/L following 72 h of fermentation in the presence of glucose and monosodium glutamate [53]. When hydrolyzed corn gluten meal, which is rich in aspartate and glutamate, was used as both a carbon and nitrogen source, *Vibrio* sp. CS1 and *Salinivibrio costicola* SH3 achieved ectoine titers of 477 mg/L and 215 mg/L, respectively, after 78 h of fermentation [28]. The soluble carbon sources used in most previous studies, including sucrose, glucose, and amino acid mixtures, are either directly metabolizable or require only simple hydrolysis. Colloidal chitin, in contrast, is an insoluble polymer that entails a more energy intensive breakdown process. Nevertheless, strain X511 produced 0.423 g/L ectoine in just 3 days, a titer on par with the best reported values from optimized systems [28]. This result suggests that strain X511 is capable of utilizing complex carbon sources. However, ectoine production declined after reaching this peak. This decrease is proposed to result from the gradual depletion of colloidal chitin, after which strain X511 may initiate the ectoine degradation pathway and use ectoine as a carbon source. In addition, the strain grew and produced ectoine on GlcNAc, the monomer of chitin, as the sole carbon source, suggesting that strain X511 can utilize chitin-derived carbon for ectoine biosynthesis via the proposed GlcNAc metabolic pathway. However, direct carbon-flow evidence that chitin-derived carbon is incorporated into ectoine was not obtained in this study, and this remains to be addressed in future work.

Selection and relative proportions of carbon and nitrogen sources play a critical role in regulating ectoine biosynthesis. *Chromohalobacter salexigens* produced the most ectoine when cultivated with 40 mM glucose and 30 mM ammonium [54], whereas *Nesterenkonia xinjiangensis* responded best to ammonium phosphate as the nitrogen source [25]. Complex nitrogen sources such as yeast extract have been shown to benefit ectoine production across various strains. Among the tested nitrogen sources, yeast extract was the most effective, and its combination with ammonium chloride resulted in an ectoine titer of 7.29 g/L [55]. Similarly, cultivated with monosodium glutamate and 0.5% yeast extract, *Halomonas venusta* DSM4743 produced 3.2 g/L ectoine in batch fermentation (10 L) and 14.7 g/L via bacterial milking [56]. Consistent with this, yeast extract enhanced ectoine production in strain X511. With 5 g/L yeast extract as the nitrogen source, the ectoine titer reached 2.172 g/L on colloidal chitin, significantly more than with inorganic nitrogen sources, suggesting that yeast extract promotes ectoine biosynthesis in this strain.

α-chitin is the most prevalent and extensively investigated polymorph of chitin, distinguished by its highly ordered and densely packed crystalline architecture [57]. Commercially available crab shell flakes, though subjected to deproteinization and demineralization, are therefore enriched in chitin and still preserve their highly crystalline and compact structure. This feature renders them more resistant to microbial breakdown than colloidal chitin, which is characterized by lower crystallinity and a looser morphology. The crystallinity of chitin plays a major role in determining its susceptibility to glycoside hydrolases. Reducing this crystallinity through physical, chemical, or microbial pretreatment has therefore become a key strategy for improving chitin accessibility and subsequent hydrolysis efficiency [58,59,60]. In this study, strain X511 degraded crystalline crab shell flakes and accumulated ectoine within 5 days. These results suggest the presence of a chitinolytic enzyme system in strain X511 and highlight its potential for converting chitinous feedstocks into value added products, although direct evidence for enzyme secretion remains to be obtained. The observed 60% weight loss is likely dominated by chitin degradation, with residual proteins and minerals possibly also contributing. FESEM, FTIR and XRD analysis further revealed the structural basis of this degradation process. In the FTIR spectrum of crab shell flakes, characteristic peaks appeared at 3440 cm^−1^ and 2929 cm^−1^, corresponding to O–H and C–H stretching vibrations [35,61]. The bands at 1700–1600 cm^−1^ were assigned to amide I C=O stretching and hydrogen bonds, the peak at 1025 cm^−1^ to asymmetric C–O–C stretching of the sugar ring [62,63], while the peak at 1557 cm^−1^ was assigned to the N–H bending and C–N stretching of amide II [64]. The absorption at 3261 cm^−1^ corresponded to N–H stretching vibrations constrained by C(2)–NH⋯O=C(7) [65]. After 7 days of treatment with strain X511, the intensities of these peaks increased significantly. The increased peak intensities suggested changes in the hydrogen bonding environment or the exposure of functional groups within the chitin structure. The enhanced signals in the O–H and C=O regions are consistent with the FTIR-based oxidative activity screening reported for BpLPMO10 on β-chitin, colloidal chitin, phosphoric acid-swollen cellulose (PASC) and microcrystalline cellulose (MCC) [66], suggesting that strain X511 may employ a similar LPMO mediated oxidative cleavage mechanism. However, this remains a proposed mechanism, as direct evidence for oxidative cleavage products was not obtained in this study. XRD analysis confirmed the reduction in crystallinity of crab shell flakes after X511 treatment. Based on the combined FESEM, FTIR and XRD results, we propose that strain X511 may exert oxidative cleavage activity against highly crystalline α-chitin during the initial fermentation phase. This modification is proposed to enhance substrate accessibility for chitinases, thereby facilitating synergistic degradation.

Microbial bioconversion of shrimp shell waste is substantially hindered by its complex structural and chemical makeup. The shell of *Fenneropenaeus chinensis* contains 40.64 ± 0.52% protein, 31.44 ± 0.01% ash and 15.15 ± 0.69% chitin [67,68]. Chitin in shrimp and crab shells is highly recalcitrant, but it can be fully degraded by the combined action of microbial chitinases, chitosanases and LPMO [69]. Strain X511 directly degraded natural shrimp shell powder and produced ectoine. FESEM, FTIR, and XRD analyses confirmed structural and compositional changes in the shrimp shell after fermentation. Consistent with these structural changes, the shrimp shell powder showed a 43.47% weight loss, reflecting the combined loss of organic and inorganic components. Among these analyses, FESEM analysis of the residues revealed flower-like crystalline aggregates, which closely resemble the hydroxyapatite nanosheet-assembled flower-like hierarchical nanostructures (NFHNs) [70]. This suggests that the aggregates may be biogenic hydroxyapatite, a possibility that requires further verification. FTIR and XRD analyses confirmed the efficient degradation of shrimp shell by strain X511. After 11 days, the chitin specific peaks at 1026, 1654, and 2929 cm^−1^ were markedly reduced, and the protein related peak at 1540 cm^−1^ disappeared [71,72,73], indicating effective decomposition of both chitin and proteins. Meanwhile, the absorption band attributed to the C–O stretching vibration of carbonate at 1409 cm^−1^ [74,75] and that attributed to the CO_3_^2−^ vibration of calcite at 874 cm^−1^ [74,76,77] were markedly enhanced (Figure 7g). The enhanced FTIR signals of carbonate and calcite suggested a relative accumulation of inorganic salts following microbial degradation of organic matter by strain X511, which was further corroborated by XRD analysis. The untreated shrimp shell powder showed characteristic chitin peaks at 9.13° and 19.19° (2θ), together with additional peaks at 39.59°, 43.34° and 47.69° (2θ) corresponding to calcite [78]. After 11 days of treatment, the chitin peaks disappeared, the calcite peaks narrowed, and the quartz peak at 29.53° (SiO_2_, ICDD No. 00-070-3755) [79] intensified markedly. Together, the FTIR and XRD results confirmed that organic degradation led to relative enrichment of inorganic salts. Moreover, FESEM and XRD jointly suggested the potential formation of hydroxyapatite, supported by the flower-like aggregates and the characteristic diffraction peaks at 31.81° and 45.48° (2θ) [80]. However, the formation of hydroxyapatite remains a proposed explanation, as direct evidence, for example, isolated hydroxyapatite characterization was not obtained. These results indicate that strain X511 facilitates organic decomposition and may also promote inorganic transformation. The production of ectoine, combined with the possible formation of biogenic hydroxyapatite, highlights the potential of this strain for the integrated valorization of shrimp shell waste into multiple high value products.

NAGase (EC 3.2.1.52) breaks the β-1,4-glycosidic bonds in N-acetylchitooligosaccharides to generate GlcNAc [81], a key step for fully depolymerizing chitin into its monomer units. Additionally, it works synergistically to improve the enzymatic breakdown of crystalline α-chitin [82]. Such a synergistic effect is well exemplified by Sohex, a NAGase derived from *Sulfuriroseicoccus oceanibius*, enhanced α-chitin conversion by 1.37-fold and 2.46-fold over the LPMOs + chitinases system and the sole chitinase system, respectively [83]. Unexpectedly, under identical conditions, the extracellular NAGase activities detected in this study were substantially lower than the chitinase activities. Nevertheless, such a discrepancy does not necessarily reflect a weak chitinolytic capability of this strain. The chitin degrading factors (CDFs) of Chitinibacter tainanensis have been shown to localize on the cell surface, with NAGase activity detected mainly in the cell debris fraction [84]. Similarly, *Paenibacillus* sp. FPU-7, a highly efficient chitin degrading bacterium, anchors a unique multi-modular chitinase (ChiW) to its cell surface, enabling efficient hydrolysis of various forms of chitin, including colloidal and crystalline chitin [85,86]. These findings suggest that cell-surface anchoring of chitinolytic enzymes and NAGase may represent a strategy employed by some microorganisms for efficient chitin degradation. Whether strain X511 adopts a similar strategy, however, remains hypothetical. SignalP 6.0 prediction revealed that the GH3 family NAGase (GM003638) in strain X511 lacks a signal peptide, indicating that it is not a secreted protein. In contrast, the GH20 family NAGase (GM000261) contains a lipoprotein signal peptide, suggesting its possible anchoring to the cell membrane. This differential localization may offer a plausible explanation for the low extracellular NAGase activity observed, and may suggest that strain X511 concentrates chitinolytic activities near the cell surface to facilitate substrate utilization. Further experiments, such as subcellular fractionation or fluorescence microscopy, are needed to confirm the membrane association of the GH20 family NAGase.

Strain X511 distinguishes itself from most wild-type strains by directly utilizing untreated shrimp shell powder for ectoine synthesis, a trait largely attributable to its predicted extracellular enzyme system, including proteases, LPMOs, chitinases, and NAGases, which are proposed to collectively depolymerize the complex chitin-protein-calcium matrix into assimilable GlcNAc. Compared with the conventional two-step process involving chemical or enzymatic pretreatment of chitin followed by microbial fermentation [87], strain X511 enables a streamlined, one-step conversion route that reduces processing costs and operational complexity. The strain achieved 2.172 g/L ectoine from colloidal chitin (Table 4), a titer comparable to or higher than those reported for some wild-type strains on glucose, and it also directly utilized crystalline α-chitin and untreated shrimp shell powder, highlighting its potential for chitinous waste biorefinery. These features suggest that strain X511 is a promising candidate for industrial application in chitinous waste valorization. This gives strain X511 a key advantage over conventional ectoine production systems, as it can directly utilize untreated chitinous feedstocks without pretreatment or exogenous enzymes. Nevertheless, further efforts are needed to enhance ectoine productivity. This may be achieved through fermentation optimization and metabolic engineering. In addition, the regulatory mechanisms governing the coordinated expression of the degradation and synthesis pathways remain to be elucidated.

Several limitations also remain. Direct carbon-flow evidence was not obtained. Quantitative chitin conversion, carbon balance, and bioreactor-scale performance also remain to be determined. Future work should address these gaps through carbon-balance analysis, quantitative chitin conversion measurements, and bioreactor-scale evaluation. Beyond this, metabolic engineering and synthetic biology could further expand the application of strain X511 to other chitinous wastes and value-added products.

## 4. Materials and Methods

### 4.1. Bacterial Strain and Culture Conditions

Strain X511 was isolated from decaying *Sargassum* by the group of Prof. Huang Shushi of Guangxi Academy of Sciences and is maintained in the Marine Culture Collection of Beibu Gulf. To prepare the seed culture, the strain was inoculated into Luria–Bertani (LB) broth and incubated at 30 °C for 18 h with shaking at 200 r/min.

The basal medium for ectoine production contained the following components per liter: 60 g NaCl, 9.7 g MgSO_4_·7H_2_O, 5 g (NH_4_)_2_SO_4_, 1 g K_2_HPO_4_, 2 g KCl, 0.2 g CaCl_2_, and 0.02 g Fe_2_SO_4_·7H_2_O. Three insoluble substrates, including colloidal chitin, crab shell flakes, and shrimp shell powder, were tested as carbon sources. Fermentations were carried out in 250 mL flasks containing 50 mL of medium, inoculated with 5% (*v*/*v*) seed culture, and incubated at 30 °C with shaking at 200 r/min.

### 4.2. Preparation of Chitin Substrates

Crab shells were purchased from Sangon Biotech (Shanghai, China; catalog No. A500659-0500). The shells were pulverized into small fragments using a grinder and then used for colloidal chitin preparation following a previously described method [88]. Shrimp shell waste was collected from fresh *Fenneropenaeus chinensis* obtained from a local supermarket in Nanning, China. The shells were rinsed, dried at 60 °C for 24 h, and ground into powder.

### 4.3. Experimental Design for Ectoine Production from Colloidal Chitin by Strain X511 

#### 4.3.1. NaCl Concentration Optimization for Ectoine Production 

The effect of NaCl concentration on ectoine production was assessed by cultivating strain X511 in a basal medium with colloidal chitin (10 g/L) as the sole carbon source. The NaCl concentration in the medium was adjusted to final concentrations of 1%, 3%, 5%, 6%, 7%, and 9% (*w*/*v*). The initial pH was adjusted to approximately 7.0 using 1 M NaOH or HCl, and the cultures were incubated for 7 days.

#### 4.3.2. Dynamic Coupling of Chitin Degradation and Ectoine Biosynthesis

Strain X511 was cultivated in a basal medium containing 6% NaCl and 10 g/L colloidal chitin as the sole carbon source to investigate the temporal dynamics of ectoine production. The fermentation was carried out over 11 d, with samples collected daily for the measurement of biomass (OD_600_), chitinase activity, and ectoine titer. β-N-acetylglucosaminidase (NAGase) activity was additionally determined at 3, 5, and 7 days of fermentation.

For OD_600_ measurement, insoluble chitin particles were first removed by a two-step differential centrifugation. The culture broth was centrifuged at 500 r/min for 2 min to sediment the chitin particles, and the resulting supernatant was transferred to a fresh tube and centrifuged at 5000 rpm for 2 min to collect the bacterial cells. The cell pellet was washed twice with 0.9% NaCl, resuspended in the same volume of 0.9% NaCl, appropriately diluted, and then measured at OD_600_. An uninoculated control was processed in parallel and used as the blank.

#### 4.3.3. GlcNAc Utilization for Ectoine Production

Strain X511 was cultivated in medium with GlcNAc (10 g/L; Shanghai Yuanye Bio-Technology Co., Ltd., Shanghai, China) as the sole carbon source to verify the downstream pathway from GlcNAc to ectoine. Biomass and ectoine titer were recorded every 12 h over 72 h.

#### 4.3.4. Optimization of Carbon and Nitrogen Sources for Ectoine Production from Colloidal Chitin 

Optimization of carbon and nitrogen sources was carried out to evaluate the potential of strain X511 for ectoine production from colloidal chitin. Colloidal chitin was tested at 10, 30, 50, and 60 g/L. With the optimal carbon source concentration determined, various nitrogen sources (with the original 5 g/L ammonium sulfate was removed), including ammonium sulfate, ammonium chloride, ammonium phosphate, ammonium oxalate, ammonium carbonate, yeast extract (Oxoid, Basingstoke, UK), and peptone (Beijing Aoboxing Bio-tech Co., Ltd., Beijing, China), were individually added at 5 g/L to evaluate their effects on ectoine production. All fermentations were conducted for 7 days.

#### 4.3.5. Ectoine Extraction via Bacterial Milking

Ectoine was extracted using the bacterial milking method [89,90]. Briefly, 1 mL of fermentation broth was centrifuged at 12,000 r/min for 2 min, and the cell pellet was resuspended in 1 mL 1% NaCl, then shaken at 30 °C and 200 r/min for 20 min, allowing intracellular ectoine to be released into the supernatant upon hypoosmotic shock. The supernatant was then collected after centrifugation for subsequent analysis. The ectoine measured in this supernatant represents the intracellular ectoine released by hypoosmotic shock, rather than ectoine secreted during fermentation or the total combined amount.

#### 4.3.6. HPLC Analysis of Ectoine

Ectoine quantification was carried out on a Waters e2695 HPLC system equipped with a PDA detector (Waters, USA), using an AR C18 column (4.6 × 250 mm, 5 μm, Horizon, UK) for separation. Isocratic elution was performed at 0.5 mL/min with 2% acetonitrile (Fisher Chemical, Pittsburgh, PA, USA) as the mobile phase, and the eluate was monitored at 210 nm. Prior to injection, the samples were appropriately diluted and filtered through 0.22 μm PTFE membranes (Tianjin Linghang Experimental Equipment Co., Ltd., Tianjin, China).

#### 4.3.7. Method Validation of Ectoine Quantification

Ectoine was quantified by HPLC using a calibration curve prepared with an ectoine standard purchased from Aladdin (Shanghai, China). Standard solutions were prepared at concentrations ranging from 0.025 to 0.25 mg/mL. The calibration curve was constructed using ectoine standards, and the regression equation was y = 6 × 10^7^x + 2.44259 × 10^5^ (R^2^ = 0.9999), where y is the peak area and x is the ectoine concentration (mg/mL). The accuracy and precision of HPLC method were evaluated by spiked recovery and repeated injections, and the results were summarized in Table 5.

The LOD and LOQ were determined by the calibration curve method and were calculated to be 2.99 and 9.07 μg/mL, respectively, using the following formula (1):

LOD = 3.3 × *S*y·x/S, LOQ = 10.0 × *S*y·x/S (1)

where *Sy·x* is standard deviation of residuals from line and S is the slope [91].

The accuracy of the method was evaluated by spiked recovery experiments at three concentration levels (0.025, 0.100, and 0.150 mg/mL), with three parallel samples at each level. The recovery was calculated using the following formula (2):

Recovery (%) = [ (*C*_spiked_ − *C*_unspiked_)/*C*_added_] × 100% (2)

where *C*_spiked_ is the ectoine concentration measured in the spiked sample, *C*_unspiked_ is the ectoine concentration measured in the corresponding unspiked sample, and *C*_added_ is the known concentration of ectoine added to the sample [92].

The precision was evaluated by six consecutive injections of one selected sample at each concentration level. The relative standard deviation (RSD) was calculated using the following formula (3):

RSD (%) = SD/Mean × 100%(3)

where SD and Mean are the standard deviation and mean of the peak areas from the six consecutive injections, respectively.

### 4.4. Enzyme Activity Assay

#### 4.4.1. Chitinase Activity Assay

Chitinase activity was measured using the 3,5-dinitrosalicylic acid (DNS; Chengdu Kelong Chemical Reagent Factory, Chengdu, China) method [93], with the fermentation supernatant serving as the crude enzyme. The reaction mixture containing 200 µL crude enzyme and 500 µL 1% (*w*/*v*) colloidal chitin in 50 mM PBS (pH 6.0), and was incubated at 40 °C for 30 min. The reaction was stopped with 750 µL DNS, followed by boiling for 10 min. After centrifugation at 5000 r/min for 2 min, the supernatant was collected and its absorbance at 540 nm was recorded, with heat inactivated enzyme as the blank. One unit (U) of enzyme activity was defined as the amount of enzyme required to liberate 1 µmol of β-N-acetylglucosamine per minute.

#### 4.4.2. β-N-Acetylglucosaminidase(NAGase) Activity Assay

NAGase activity was assayed using the p-nitrophenol (pNP) method [83] with minor modifications, and the fermentation supernatant was used as the crude enzyme. 50 µL of crude enzyme was mixed with 50 µL of 1 mM pNP-GlcNAc (Macklin, Shanghai, China) in 50 mM PBS (pH 6.0), and the mixture was incubated at 40 °C for 10 min. The reaction was terminated by adding 200 µL of 0.2 M NaOH. After 10 min, the absorbance of the liberated pNP at 410 nm was measured, with heat-inactivated enzyme as the blank. One unit (U) of NAGase activity was defined as the amount of enzyme required to release 1 µmol of p-nitrophenol per min.

### 4.5. Potential of Strain X511 for Ectoine Production from Chitinous Waste

The ability of strain X511 to utilize different chitinous substrates was evaluated by cultivation with commercial crab shell flakes for 7 days and raw shrimp shell powder for 11 days. Samples were collected every 2 days to monitor biomass (OD_600_), ectoine titer, and chitinase activity. NAGase activity was additionally determined at 3, 5, and 7 days of fermentation for both substrates.

#### 4.5.1. Determination of Substrate Weight Loss Rate

Residual substrates were collected by centrifugation at 500 r/min for 10 min after fermentation, washed three times with deionized water, and lyophilized to constant weight. Untreated substrates were processed in parallel as controls. The weight loss rate was calculated using the following formula (4):

Weight loss (%) = [(*W*_0_ − *W*_1_) / *W*_0_] × 100% (4)

where *W*_0_ and *W*_1_ represent the dry weights of the untreated and strain treated residual substrates, respectively.

#### 4.5.2. SEM, FTIR, and XRD Analyses

Substrate residues were sputter-coated with gold (40 mA, 60 s) and examined by field emission scanning electron microscopy (FE-SEM) using either a Quattro S (Thermo Fisher Scientific, Waltham, MA, USA) or a Supra55 Sapphire (Carl Zeiss, Oberkochen, Germany) instrument. Imaging was performed at accelerating voltages of 10.0–20.0 kV, with a magnification of 20,000×. FTIR spectra were recorded on a Nicolet iS10 spectrometer (Thermo Fisher Scientific, Madison, WI, USA) over the range of 4000–400 cm^−1^ at a resolution of 2 cm^−1^. X-ray diffraction (XRD) patterns were collected on a Rigaku Ultima IV diffractometer (Rigaku, Tokyo, Japan) at 40 kV and 40 mA, with a scanning range of 5° to 60° (2θ), a step size of 0.02°, and a scan rate of 5°/min.

### 4.6. Genome Analyses

Whole genome sequencing of strain X511 was outsourced to Novogene Bioinformatics Technology Co., Ltd. (Beijing, China). Gene function annotation was performed using the NR, GO, COG, and KEGG databases. Signal peptides were predicted using SignalP 6.0, and conserved domains were identified through the NCBI Conserved Domain Database (CDD). Putative biosynthetic gene clusters for secondary metabolites were identified using antiSMASH (https://antismash.secondarymetabolites.org (accessed on 10 October 2026)). ChiPlot (https://www.chiplot.online/ (accessed on 10 October 2026)) was employed for the visualization of protein domain architectures and gene cluster organizations.

### 4.7. Statistical Analysis

All fermentation experiments were performed in triplicate (*n* = 3 biologically independent replicates). Statistical differences among groups were analyzed by one-way ANOVA using SPSS 25.0, followed by Tukey’s post-hoc test. A *p* value < 0.05 was considered statistically significant. Data are presented as mean ± standard deviation (SD) of three independent replicates. Different lowercase letters (a, b, c) in the figures indicate significant differences among groups (*p* < 0.05).

## 5. Conclusions

Strain X511 was characterized as a wild-type isolate capable of producing ectoine while utilizing and degrading chitinous biomass. Genomic analysis revealed a complete ectoine gene cluster (*ectABC-ask*) along with genes encoding GH18/GH19 chitinases, GH20/GH3 NAGases, and LPMO10s, which are proposed to constitute the genetic basis for this function. The strain produced ectoine directly from colloidal chitin, crab shell chips, and raw shrimp shell powder without exogenous enzymes, reaching a titer of 2.172 g/L on colloidal chitin. The degradation of crystalline chitin and shrimp shell matter was corroborated by FESEM, XRD, and FTIR analyses. A reduction in shell weight was also observed after fermentation. In addition, hydroxyapatite-like crystals were tentatively observed in the shrimp shell residues following X511 treatment, a finding that awaits further confirmation. Collectively, these results indicate that strain X511 holds promise for the valorization of chitinous waste. However, direct carbon-flow evidence was not obtained, and the proposed enzyme functions require further confirmation. Future work should address these gaps. In addition, metabolic engineering and synthetic biology approaches could further expand the application of strain X511 to other chitinous wastes and value-added products.

## Figures and Tables

**Table 1:** The annotation of protein coding gene in strain X511.

Annotation in Database	NO. of Genes	%
NR	4620	97.61
SwissProt	2257	47.69
KEGG	2833	59.86
COG	3552	75.05
GO	3308	69.89

**Table 2:** Summary of CAZy family distribution.

CAZy_Class	Number of Gene
CBM	25
CE	5
GH	46
GT	26
PL	9

**Table 3:** NAGase activities during ectoine production from various chitin substrates by strain X511.

Substrates (10 g/L)	NAGase Activity (mU/mL)		
	3 days	5 days	7 days
Colloidal chitin	10.47 ± 0.87 c	15.11 ± 0.75 b	18.46 ± 0.54 a
Crab shell flakes	5.47 ± 0.78 b	10.07 ± 0.94 a	11.08 ± 0.72 a
Shrimp shell powder	3.84 ± 0.61 a	4.78 ± 0.65 a	4.23 ± 0.30 a

Different lowercase letters indicate significant differences (*p* < 0.05).

**Table 4:** Ectoine production by wild-type bacteria.

Strain	Substrate	Substrate Type	NaCl%	Fermentation	Ectoine (g/L)	Reference
*Brevibacterium* sp. JCM 6894	Glucose	Soluble, purified	1.76 to 11.7	Shake flask	0.8	[24]
*Nesterenkonia xinjiangensis*	Sucrose	Soluble, purified	8.13	Shake flask	1.38	[25]
*Halomonas salina* DSM 5928	Monosodium glutamate	Soluble, purified	2.93	Batch fermentation	6.9	[27]
*Halomonas campaniensis* XH26	Sodium L-glutamate, casein enzymatic hydrolysate	Soluble, purified/hydrolyzed	5	Shake flask	0.51	[37]
*Halomonas ventosae* DL7	Bactopeptone, Bacto yeast extract	Soluble, purified/complex	17.55	Shake flask	0.4048	[38]
*Halomonas elongata* DSM 2581T	glucose		8	Batch fermentation	12.91	[39]
	Penicillin fermentation fungal residue hydrolysate	Soluble, hydrolyzed	8	Shake flask	3.96	[40]
	Penicillin fermentation fungal residue hydrolysate, corn steep liquor	Soluble, hydrolyzed/complex	8 to 12	Fed-batch fermentation	15.84	[40]
X511	Colloidal chitin	Insoluble, polymeric	6	Shaking flask	2.172	This study
	Commercial crab shell		6	Shaking flask	0.259	This study
	shrimp shell powder		6	Shaking flask	0.181	This study

**Table 5:** Precision and recovery of the HPLC method for ectoine quantification.

Concentration (mg/mL)	Recovery (%)	RSD (%)
0.025	93.31	0.34
0.100	96.65	0.55
0.150	98.24	0.43

## Data Availability

The original contributions presented in this study are included in the article. The genomic data have been deposited in NCBI, and the accession numbers are provided in the main text. Further inquiries can be directed to the corresponding authors.

## References

[1] Salmon S., Hudson S.M. (1997). Crystal Morphology, Biosynthesis, and Physical Assembly of Cellulose, Chitin, and Chitosan. J. Macromol. Sci..

[2] Verma M.L., Kumar S., Das A., Randhawa J.S., Chamundeeswari M. (2020). Chitin and chitosan-based support materials for enzyme immobilization and biotechnological applications. Environ. Chem. Lett..

[3] Hossin M.A., Shaqsi N.H.K.A., Touby S.S.J.A., Al Sibani M.A. (2021). A review of polymeric chitin extraction, characterization, and applications. Arab. J. Geosci..

[4] Yan N., Chen X. (2015). Sustainability: Don’t waste seafood waste. Nature.

[5] Venugopal V. (2021). Valorization of Seafood Processing Discards: Bioconversion and Bio-Refinery Approaches. Front. Sustain. Food Syst..

[6] Zhu H., Chen S., Xue J., Wang X., Yang T., He J., Luo Y. (2025). Advances and challenges in green extraction of chitin for food and agriculture applications: A review. Int. J. Biol. Macromol..

[7] Iglesias-Montes M.L., Soccio M., Siracusa V., Gazzano M., Lotti N., Cyras V.P., Manfredi L.B. (2022). Chitin Nanocomposite Based on Plasticized Poly(lactic acid)/Poly(3-hydroxybutyrate) (PLA/PHB) Blends as Fully Biodegradable Packaging Materials. Polymers.

[8] Hui C., Jiang H., Liu B., Wei R., Zhang Y., Zhang Q., Liang Y., Zhao Y. (2020). Chitin degradation and the temporary response of bacterial chitinolytic communities to chitin amendment in soil under different. Sci. Total Environ..

[9] Li Y., Qiu D., Zhuang X., Pei Z., Li T., Deng S. (2025). Preparation and Paclitaxel-Loading of Regenerated Chitin Nanoparticles. Polymers.

[10] Shamshina J.L., Berton P. (2020). Use of Ionic Liquids in Chitin Biorefinery: A Systematic Review. Front. Bioeng. Biotechnol..

[11] Tsurkan M.V., Voronkina A., Khrunyk Y., Wysokowski M., Petrenko I., Ehrlich H. (2021). Progress in chitin analytics. Carbohydr. Polym..

[12] Mao X., Guo N., Sun J., Xue C. (2017). Comprehensive utilization of shrimp waste based on biotechnological methods: A review. J. Clean. Prod..

[13] Lopes C., Antelo L.T., Franco-Uría A., Alonso A.A., Pérez-Martín R. (2018). Chitin production from crustacean biomass: Sustainability assessment of chemical and enzymatic processes. J. Clean. Prod..

[14] Hammami I., Siala R., Jridi M., Ktari N., Nasri M., Triki M.A. (2013). Partial purification and characterization of chiIO8, a novel antifungal chitinase produced by Bacillus cereus IO8. J. Appl. Microbiol..

[15] Lu D.-L., Zhang M.-S., Deng J.-J., Lu W.-J., Yang Z.-D., Li Z.-W., Chen Y.-C., Luo X.-C. (2023). Highly efficient shrimp shell recovery by solid-state fermentation with Streptomyces sp. SCUT-3. Chem. Eng. J..

[16] Pan D., Xiao P., Li F., Liu J., Zhang T., Zhou X., Zhang Y. (2024). High Degree of Polymerization of Chitin Oligosaccharides Produced from Shrimp ShellWaste by Enrichment Microbiota Using Two-Stage Temperature-Controlled Technique of Inducing Enzyme Production and Metagenomic Analysis of Microbiota Succession. Mar. Drugs.

[17] Nayak P.K., Goode M., Chang D.P., Rajagopal K. (2020). Ectoine and Hydroxyectoine Stabilize Antibodies in Spray-Dried Formulations at Elevated Temperature and during a Freeze/Thaw Process. Biomed. Res. Int..

[18] Sajjad W., Qadir S., Ahmad M., Rafiq M., Hasan F., Tehan R., McPhail K.L., Shah A.A. (2018). Ectoine: A compatible solute in radio-halophilic Stenotrophomonas sp. WMA-LM19 strain to prevent ultraviolet-induced protein damage. J. Appl. Microbiol..

[19] Andrew M., Duc-Huy N., Neil B., Jared J. (2013). The active natural anti-oxidant properties of chamomile, milk thistle, and halophilic bacterial components in human skin in vitro. J. Drugs Dermatol. JDD.

[20] Mathumai K., Hee K.S., Koyeli G., Beum P.C. (2008). Small stress molecules inhibit aggregation and neurotoxicity of prion peptide 106-126. Biochem. Biophys. Res. Commun..

[21] Andreas B., Anja H., Anna R., Ralph M. (2021). Ectoine in the treatment of irritations and inflammations of the eyes surface. BioMed Res. Int..

[22] Klaus U., Ursula K., Ulrich S., Andrea A., Andreas B., Sabine S., Alessandra M., Tamara S., Stefanie K., Jean K. (2016). Reduction of neutrophilic lung inflammation by inhalation of the compatible solute ectoine: A randomized trial with elderly individuals. Int. J. Chronic Obstr. Pulm. Dis..

[23] Strong P.J., Kalyuzhnaya M., Silverman J., Clarke W.P. (2016). A methanotroph-based biorefinery: Potential scenarios for generating multiple products from a single fermentation. Bioresour. Technol..

[24] Nagata S., Wang Y.B. (2001). Accumulation of Ectoine in the Halotolerant Brevibacterium sp. JCM 6894. J. Biosci. Bioeng..

[25] Orhan F., Ceyran E.g., Akincioğlu A. (2023). Optimization of ectoine production from Nesterenkonia xinjiangensis and one-step ectoine purification. Bioresour. Technol..

[26] Xu S., Zhang B., Chen W., Ye K., Shen J., Liu P., Wu J., Wang H., Chu X. (2023). Highly efficient production of ectoine via an optimized combination of precursor metabolic modules in Escherichia coli BL21. Bioresour. Technol..

[27] Zhang L., Lang Y., Nagata S. (2009). Efficient production of ectoine using ectoine-excreting strain. Extremophiles.

[28] Omara A.M.A., Sharaf A.E.-M.M., El-Hela A.A., Shahin A.A.M., El-Bialy H.A.A., El-Fouly M.Z. (2020). Optimizing ectoine biosynthesis using response surface methodology and osmoprotectant applications. Biotechnol. Lett..

[29] Choi Y., Jung W., Lee H., Cho S., Lee J. (2024). Methylotuvimicrobium alcaliphilum 20Z based ectoine production from various industrial CH4 sources: Cultivation optimization and comprehensive analysis. Chem. Eng. J..

[30] Guo S., Li C., Su Y., Huang X., Zhang C., Dai Y., Ji Y., Fu R., Zheng T., Fei Q. (2025). Scalable Electro-Biosynthesis of Ectoine from Greenhouse Gases. Angew. Chem. Int. Ed..

[31] Holt J.D., Miller K.A., Hunter O.F., Zhang E., Hinbest A.J., Gerace E., Olson R., Kadouri D.E., Nadell C.D. (2026). Predator–prey dynamics of Vibrio cholerae on chitin suggest an alternative mode of biofilm formation in marine snow conditions. ISME J..

[32] Hunt D.E., Gevers D., Vahora N.M., Polz M.F. (2008). Conservation of the Chitin Utilization Pathway in the Vibrionaceae. Appl. Environ. Microbiol..

[33] Xu C., Xiong Y., Wang Q., Fang F., Wang J., Huang S., Xu J., Peng Y., Xie C. (2025). Isolation of salt-tolerant Vibrio alginolyticus X511 for efficient co-production of 2,3-butanediol and alginate lyase from Laminaria japonica. Int. J. Biol. Macromol..

[34] Xu C., Xiong Y., Lu M., Liao W., Zhang Y., Huang S. (2017). Screening and Identification of a Marine Alginate-degrading Bacteriumand the Utilization Capacity of Polysaccharide. Biotechnol. Bull..

[35] Wei G., Zhang A., Chen K., Ouyang P. (2017). Enzymatic production of N-acetyl-D-glucosamine from crayfish shell wastes pretreated via high pressure homogenization. Carbohydr. Polym..

[36] Wu D., Dan M., Kao M.-R., Li Y., Song J., Zheng Y., Zhao G., Hsieh Y.S.Y., Wang D. (2025). The Functional Characterization of an AA10 Lytic Polysaccharide Monooxygenase from Saccharophagus degradans 2-40T for Enhanced Chitin Biodegradation. Foods.

[37] Wang Z., Li Y., Gao X., Xing J., Wang R., Zhu D., Shen1 G. (2023). Comparative genomic analysis of Halomonas campaniensis wild-type and ultraviolet radiation-mutated strains reveal genomic differences associated with increased ectoine production. Int. Microbiol..

[38] Zhu D., Niu L., Wang C., Nagata S. (2007). Isolation and characterisation of moderately halophilic bacterium Halomonas ventosae DL7 synthesizing ectoine as compatible solute. Ann. Microbiol..

[39] Yu J., Wang Z., Wang J., Mohisn A., Liu H., Zhang Y., Zhuang Y., Guo M. (2022). Physiological metabolic topology analysis of Halomonas elongata DSM 2581T in response to sodium chloride stress. Biotechnol. Bioeng..

[40] Liu Y., Zhang S., Zhang G., Zhang Y., Yang C., Wang Z., Yu J., Wu S. (2025). Resource utilization of penicillin fermentation fungi residue for ectoine production by Halomonas elongata. Bioresour. Technol..

[41] Ikegam T., Okada T., Hashimoto M., Seino S., Watanabe T., Shirakawa M. (2000). Solution Structure of the Chitin-binding Domain of Bacillus circulans WL-12 Chitinase A. J. Biol. Chem..

[42] Zhao H., SuJianan H., Dong S., Mao X. (2024). Bioconversion of α-Chitin by a Lytic Polysaccharide Monooxygenase OsLPMO10A Coupled with Chitinases and the Synergistic Mechanism Analysis. J. Agric. Food Chem..

[43] Vermaas J.V., Crowley M.F., Beckham G.T., Payne C.M. (2015). Effects of Lytic Polysaccharide Monooxygenase Oxidation on Cellulose Structure and Binding of Oxidized Cellulose Oligomers to Cellulases. J. Phys. Chem. B.

[44] Ghosh S., Rao H., Sengupta M., Bhattacharya S.K., Datta A. (2011). Two gene clusters co-ordinate for a functional N-acetylglucosamine catabolic pathway in Vibrio cholerae. Mol. Microbiol..

[45] Yamano N., Oura N., Wang J., Fujishima S. (1997). Cloning and Sequencing of the Genes for N-Acetylglucosamine Use That Construct Divergent Operons (nagE-nagA q from Vibrio cholerae Non-Ot. Biosci. Biotechnol. Biochem..

[46] Stöveken N., Pittelkow M., Sinne T., Jensen R.A., Heider J., Bremer E. (2011). A Specialized Aspartokinase Enhances the Biosynthesis of the Osmoprotectants Ectoine and Hydroxyectoine inPseudomonas stutzeri A1501. J. Bacteriol..

[47] Zheng L., Chen X., Yuan L., Wu J., Yao J. (2025). Metabolic Control for High-Efficiency Ectoine Synthesis in Engineered Escherichia coli. ACS Synth. Biol..

[48] Han J., Gao Q.-X., Zhang Y.-G., Li L., Mohamad O.A.A., Rao M.P.N., Xiao M., Hozzein W.N., Alkhalifah D.H.M., Yong Tao 2 W.-J.L. (2018). Transcriptomic and Ectoine Analysis of Halotolerant Nocardiopsis gilva YIM 90087T Under Salt Stress. Extrem. Microbiol..

[49] Wood J.M. (2015). Bacterial responses to osmotic challenges. J. Gen. Physiol..

[50] Gregory G.J., Morreale D.P., Boyd E.F. (2020). CosR Is a Global Regulator of the Osmotic Stress Response with Widespread Distribution among Bacteria. Appl. Environ. Microbiol..

[51] Zhou N., Wilkes R.A., Chen X., Teitel K.P., Belgrave J.A., Beckham G.T., Werner A.Z., Yu Y., Aristilde L. (2025). Quantitative decoding of coupled carbon and energy metabolism in Pseudomonas putida for lignin carbon utilization. Commun. Biol. Vol..

[52] Siqueiraht G.M.V.d., Srinivasan A., Chen Y., Gin J.W., Petzold C.J., Lee T.S., Guazzaroni M.-E., Eng T., Mukhopadhyay A. (2025). Alternate routes to acetate tolerance lead to varied isoprenol production from mixed carbon sources in Pseudomonas putida. Appl. Environ. Microbiol..

[53] Haoxin L., Shanshan H., Zonghao Z., Yongzhen L., Rong W., Rui H., Derui Z. (2026). Metabolic engineering coupled with response surface methodology for optimization of ectoine synthesis in Halomonas. Acta Microbiol. Sin..

[54] Salar-García M.J., Bernal V., Pastor J.M., Salvador M., Argandoña M., Nieto J.J., Vargas C., Cánovas M. (2017). Understanding the interplay of carbon and nitrogen supply for ectoines production and metabolic overflow in high density cultures of Chromohalobacter salexigens. Microb. Cell Factories.

[55] Hao Z., Zhong L., Ming Z., Yanqin M., Zhengshan L., Sha L., Hong X. (2022). Metabolic Engineering of Escherichia coli for Ectoine Production With a Fermentation Strategy of Supplementing the Amino Donor. Front. Bioeng. Biotechnol..

[56] Zheng X., Ma H., Yan X., Zhang L. (2010). Ectoine Synthesis and Release Under Osmotic Shock in Halomonas venusta DSM4743. Microbiol. China.

[57] Minke R., Blackwell J. (1978). The structure of α-chitin. J. Mol. Biol..

[58] He Y., Zhang C., Zhang X., Li Y., Zhang Q. (2024). Plasma-activated water improves the accessibility of chitinase to chitin by decreasing molecular weight and breaking crystal structure. Carbohydr. Res..

[59] Sivaramakrishna D., Bhuvanachandra B., Mallakuntla M.K., Das S.N., Ramakrishna B., Podile A.R. (2020). Pretreatment with KOH and KOH-urea enhanced hydrolysis of α-chitin by an endo-chitinase from Enterobacter cloacae subsp. cloacae. Carbohydr. Polym..

[60] Kumar M., Madhuprakash J., Balan V., Singh A.K., Vivekanand V., Pareek N. (2021). Chemoenzymatic production of chitooligosaccharides employing ionic liquids and Thermomyces lanuginosus chitinase. Bioresour. Technol..

[61] Yang Y., Chang Y., Wu Y., Liu H., Liu Q., Kang Z., Wu M., Yin H., Duan J. (2021). A homogeneous polysaccharide from Lycium barbarum: Structural characterizations, anti-obesity effects and impacts on gut microbiota. Int. J. Biol. Macromol..

[62] Yea S., Jina W., Huanga Q., Hua Y., Lia Y., Lia J., Lia B. (2017). Da-KGM based GO-reinforced FMBO-loaded aerogels for efficientarsenic removal in aqueous solution. Int. J. Biol. Macromol..

[63] Brugnerottoa J., Lizardib J., Goycooleab F.M., ArguÈelles-Monalc W., DesbrieÁresa J., Rinaudoa M. (2001). An infrared investigation in relation with chitin and chitosan characterization. Polymer.

[64] Tan Y.N., Lee P.P., Chen W.N. (2020). Microbial extraction of chitin from seafood waste using sugars derived from fruit waste-stream. AMB Express.

[65] Wu Y., Sasaki T., Irie S., Sakurai K. (2008). A novel biomass-ionic liquid platform for the utilization of native chitin. Polymer.

[66] Hong H., Wang X., Huang L., Yang D., Guo H., Shao X., Chen H., Pang Q., Pan L. (2025). Characterization of BpLPMO10 from Bacillus paralicheniformis: A bi-functional AA10 LPMO with chitin-preferential activity and strong exo-enzyme synergy. Int. J. Biol. Macromol..

[67] Synowiecki J., Al-Khateeb N.A.A.Q. (2000). The recovery of protein hydrolysate during enzymatic isolation of chitin from shrimp Crangon crangon processing discards. Food Chem..

[68] Tian Y., Yin J., Ma T., Yan W., Zhu H., Sun H. (2025). Study on electrostatic separation technology and characterization of shrimp shell protein. Sep. Purif. Technol..

[69] Jiang W.-X., Li P.-Y., Chen X.-L., Zhang Y.-S., Wang J.-P., Wang Y.-J., Sheng Q., Sun Z.-Z., Qin Q.-L., Ren X.-B. (2022). A pathway for chitin oxidation in marine bacteria. Nat. Commun..

[70] Zhao X.Y., Zhu Y.J., Chen F., Lu B.Q., Wu J. (2013). Nanosheet-assembled hierarchical nanostructures of hydroxyapatite: Surfactant-free microwavehydrothermal rapid synthesis, protein/DNA adsorption and pH-controlled release. Cryst. Eng. Commun..

[71] Maruthiah T., Somanath B., Immanuel G., Palavesam A. (2017). Investigation on Production and Purification of Haloalkalophilic Organic Solvent Tolerant Protease from Marine Shell Waste and its Bioconversion to Chitin by Aquatic Bacillus sp. APCMST-CS4. Waste Biomass Valoriz..

[72] Mohammed M.H., Williams P.A., Tverezovskaya O. (2013). Extraction of chitin from prawn shells and conversion to low molecular mass chitosan. Food Hydrocoll..

[73] Maruthiaha T., Palavesam A. (2017). Characterization of haloalkalophilic organic solvent tolerant protease for chitin extraction from shrimp shell waste. Int. J. Biol. Macromol..

[74] Mikkelsen A., Engelsen S.B., Hansen H.C.B., Larsen O., Skibsted L.H. (1997). Calcium carbonate crystallization in the α-chitin matrix of the shell of pink shrimp, Pandalus borealis, during frozen storage. J. Cryst. Growth.

[75] Miller F.A., Wilkins C.H. (1952). Infrared Spectra and Characteristic Frequencies of Inorganic Ions. Anal. Chem..

[76] Fowler B.O. (1973). Infrared studies of apatites. I. Vibrational assignments for calcium, strontium, and barium hydroxyapatites utilizing isotopic substitution. Inorg. Chem..

[77] Rahman M.A., Halfar J. (2014). First evidence of chitin in calcified coralline algae: New insights into the calcification process of Clathromorphum compactum. Sci. Rep..

[78] Gbenebor O.P., Adeosun S.O., Lawal G.I., Jun S., Olaleye S.A. (2017). Acetylation, crystalline and morphological properties of structural polysaccharide from shrimp exoskeleton. Eng. Sci. Technol. Int. J..

[79] Okuda R., Komatsu K., Nakamura A., Ito O., Nambu K., Saitoh H. (2019). Evaluation of released amount of hydrogen after high pressure hydrogen loading in carbonate. Results Eng..

[80] Bui V.X., Vo M.Q., Nguyen T.A., Bui H.T. (2019). Investigation of Bioactive Glass-Ceramic 60SiO2-30CaO-10P2O5 Prepared by Hydrothermal Method. Adv. Mater. Sci. Eng..

[81] Yang S., Song S., Yan Q., Fu X., Jiang Z., Yang X. (2014). Biochemical Characterization of the First Fungal Glycoside Hydrolyase Family 3 β-N-Acetylglucosaminidase from Rhizomucor miehei. J. Agric. Food Chem..

[82] Han S., Xue Y., Yan Q., Jiang Z., Yang S. (2024). Development of a two-enzyme system in Aspergillus niger for efficient production of N-acetyl-β-D-glucosamine from powdery chitin. Bioresour. Technol..

[83] Gao J., Dong H., Zhang P., Gao K., Su H., Sun J., Mao X. (2025). Efficient Bioconversion of Crystalline α-Chitin to N-Acetyl-Dglucosamine Driven by a Novel β-N-Acetylglucosaminidase and the Promotion Mechanism Analysis. J. Agric. Food Chem..

[84] Chen J.-K., Shen C.-R., Yeh C.-H., Fang B.-S., Huang T.-L., Liu C.-L. (2011). *N*-Acetyl Glucosamine Obtained from Chitin by Chitin Degrading Factors in *Chitinbacter tainanesis*. Int. J. Mol. Sci..

[85] Itoh T. (2021). Structures and functions of carbohydrate-active enzymes of chitinolytic bacteria Paenibacillus sp. str.FPU-7. Biosci. Biotechnol. Biochem..

[86] Itoh T., Sugimoto I., Hibi T., Suzuki F., Matsuo K., Fujii Y., Taketo A., Kimoto H. (2014). Overexpression, purification, and characterization of Paenibacillus cellsurface-expressed chitinase ChiW with two catalytic domains. Biosci. Biotechnol. Biochem..

[87] Liang B., Song W., Liu S., Li K., Yu H., Li P., Xing R. (2025). Improving the Performance of Chitin Bioprocessing: Pretreating Chitin and Optimizing Active Enzyme. Polym. Rev..

[88] Subramanian K., Sadaiappan B., Aruni W., Kumarappan A., Thirunavukarasu R., Srinivasan G.P., Bharathi S., Nainangu P., Renuga P.S., Elamaran A. (2020). Bioconversion of chitin and concomitant production of chitinase and N-acetylglucosamine by novel Achromobacter xylosoxidans isolated from shrimp waste disposal area. Sci. Rep..

[89] Sauer T., Galinski E.A. (1998). Bacterial milking: A novel bioprocess for production of compatible solutes. Biotechnol. Bioeng..

[90] Rodero M.d.R., Carmona-Martínez A.A., Martínez-Fraile C., Herrero-Lobo R., Rodríguez E., García-Encina P.A., Peña M., Munoz R. (2023). Ectoines production from biogas in pilot bubble column bioreactors and their subsequent extraction via bio-milking. Water Res..

[91] More S.J., Tandulwadkar S.S., Nikam A.R., Rathore A.S., Sathiyanarayanan L., Mahadik K.R. (2012). Application of HPLC for the Simultaneous Determination of Paracetamol, Chlorzoxazone, and Nimesulide in Pharmaceutical Dosage Form. ISRN Chromatogr..

[92] Usama M., Naz S., Satti S., Ayyub A., Khan H.K., Alhidary I.A., Ülger İ., Abudabos A., Rossi L., Ahmad S. (2026). Comparative effects of Capsicum annuum selenium nanoparticles and sodium selenite on growth, antioxidant activity, bioaccumulation, and DNA damage in quails. Poult. Sci..

[93] Miller G.L. (1959). Use of dinitrosalicylic acid reagent for determination of reducing sugar. Anal. Chem..

